# Early Chemistry of Nicotine Degradation in Heat-Not-Burn Smoking Devices and Conventional Cigarettes: Implications for Users and Second- and Third-Hand Smokers

**DOI:** 10.1021/acs.jpca.1c01650

**Published:** 2021-04-09

**Authors:** Javier E. Chavarrio Cañas, M. Monge-Palacios, E. Grajales-González, S. Mani Sarathy

**Affiliations:** Clean Combustion Research Center (CCRC), Physical Science and Engineering (PSE) Division, King Abdullah University of Science and Technology (KAUST), Thuwal 23955-6900, Saudi Arabia

## Abstract

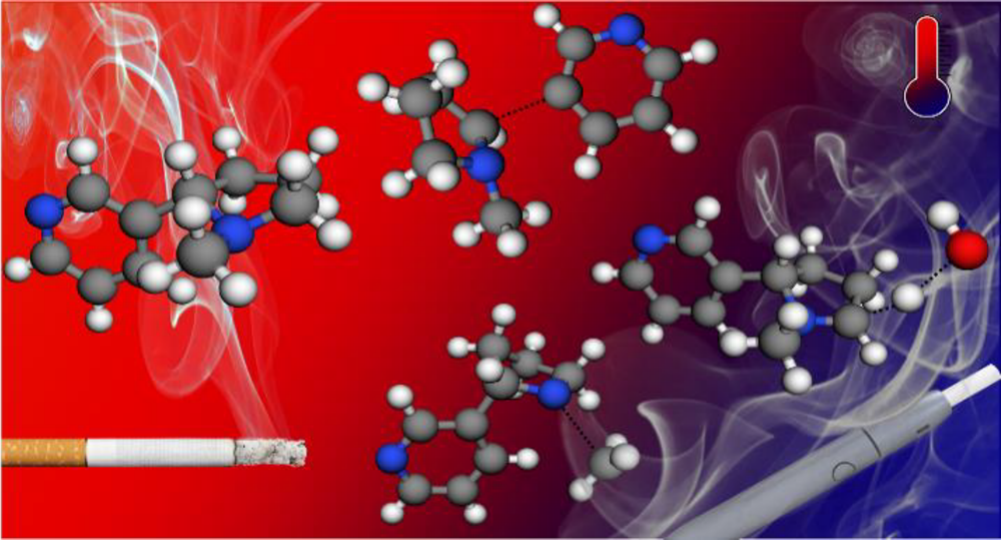

Nicotine exposure results in health risks not only for smokers but also for second- and third-hand smokers. Unraveling nicotine’s degradation mechanism and the harmful chemicals that are produced under different conditions is vital to assess exposure risks. We performed a theoretical study to describe the early chemistry of nicotine degradation by investigating two important reactions that nicotine can undergo: hydrogen abstraction by hydroxyl radicals and unimolecular dissociation. The former contributes to the control of the degradation mechanism below 800 K due to a non-Arrhenius kinetics, which implies an enhancement of reactivity as temperature decreases. The latter becomes important at higher temperatures due to its larger activation energy. This change in the degradation mechanism is expected to affect the composition of vapors inhaled by smokers and room occupants. Conventional cigarettes, which operate at temperatures higher than 1000 K, are more prone to yield harmful pyridinyl radicals via nicotine dissociation, while nicotine in electronic cigarettes and vaporizers, with operating temperatures below 600 K, will be more likely degraded by hydroxyl radicals, resulting in a vapor with a different composition. Although low-temperature nicotine delivery devices have been claimed to be less harmful due to their nonburning operating conditions, the non-Arrhenius kinetics that we observed for the degradation mechanism below 873 K suggests that nicotine degradation may be more rapidly initiated as temperature is reduced, indicating that these devices may be more harmful than it is commonly assumed.

## 1 Introduction

Cigarette smoke causes more than 8 million of fatalities per year worldwide, with ∼86% of deaths owing to direct use of tobacco and the remaining to exposure to second-hand smoke (inhalation in the presence of a smoker) and third-hand smoke (inhalation close to surfaces previously exposed to cigarette smoke).^1,2^ Tobacco smoke is a complex matrix of more than 3800 harmful species such as free radicals, heavy metals,^3^ and organic compounds.^4^ The effect of free radicals and toxic chemicals over health has been documented, attributing them an active role in development and progression of multiple ailments including cardiovascular and pulmonary disorders, asthma, and cancer.^5^ People continue smoking tobacco due to addiction to nicotine,^6^ which is the predominant alkaloid in tobacco leaves.^5,7^

Nicotine is frequently used for tobacco smoke tracking in indoor environments.^8^ After smoking, the nicotine molecules released to the environment deposit on indoor surfaces such as furniture, walls, and skin^9−11^ and reemit progressively, leading to continuous indoor exposure to nicotine. Subsequently, nicotine reacts with indoor oxidants either in the gas phase or on surfaces, producing more toxic and carcinogenic compounds such as nitrosamines.^12,13^ Undoubtedly, nicotine compromises not only the health of smokers but also that of second- and third-hand smokers even once the cigarette is extinguished.

Understanding the mechanism and kinetics of nicotine degradation (pyrolysis and oxidation) is vital to evaluate the exposure to harmful species by smokers and room occupants. Indoor oxidation of organic compounds is carried out by oxidants like ozone, hydroxyl radicals (OH), and nitrate radicals.^14^ Nonetheless, OH is recognized as the most important oxidant in the troposphere due to its high reactivity and abundance.^15^ Outdoor and indoor OH concentrations of 2 × 10^6^ and 5 × 10^5^ molecules cm^–3^, respectively, have been reported,^16,17^ but even higher indoor concentrations (10^7^ molecules cm^–3^) can be found due to photolysis of nitrous acid during periods of intense sunlight^17^ or due to the use of cleaning products.^18^ Borduas found that desorbed nicotine is oxidized by OH, forming indoor toxic substances such as isocyanic acid, formamide, acetaldehyde, and acetonitrile.^19^ Borduas *et al*. also measured the overall rate constant for the reaction between nicotine and OH to be 8.38 × 10^–11^ cm^3^ molecule^–1^ s^–1^ at 298 K^2^ and concluded that removal of nicotine by OH may compete with surface absorption and heterogeneous chemistry. However, rate constants were not reported at other temperatures that might be relevant to indoor pollutant formation, and their experiment does not indicate the relative importance of each of the different reaction pathways between nicotine and OH. This information is required to estimate the prominence of the pollutants formed in nicotine degradation.

The unimolecular degradation of nicotine has been also investigated.^7,20^ Kibet *et al.*(20) measured the formation of pyridine in commercial cigarettes and proposed a pathway for the formation of pyridinyl radicals, and ultimately pyridine, via C–C scission of nicotine. The formation mechanism of pyridinyl radicals is of interest due to its high reactivity toward DNA, lipids, and microphages, with serious health implications.^21−24^ Kurgat *et al.*(7) described pathways for the thermal degradation of nicotine and the different harmful radicals that can be formed but did not report rate constants. The operating temperatures of different smoking devices such as conventional and electronic cigarettes and vaporizers may affect the composition of their vapors, justifying the need for a kinetic study of nicotine dissociation across a wide temperature range.

New electronic devices such as IQOS are gaining attention due to their low-temperature operating conditions that prevent the combustion of tobacco; these devices operate at temperatures below 350 °C, while conventional or burning cigarettes operate at around 900 °C and thus release a more toxic aerosol.^25−27^ Nevertheless, recent works found additional chemicals in the IQOS aerosol that are not typically found in studies of targeted compounds.^4,28^ Additionally, electron spin resonance experiments have been recently performed by Bitzer *et al.*(29) and Shein and Jeschke,^30^ who determined that heat-not-burn devices produce significantly less gas-phase radicals than conventional cigarettes; however, these authors did not report which specific radicals are formed.

It is evident that more studies are necessary to unravel the degradation chemistry of nicotine under different conditions of temperature and pressure in order to assess its impact on the health of smokers and second- and third-hand smokers. In this work, we used robust theoretical methods to calculate the rate constants of the hydrogen abstraction reactions of nicotine by OH and those of nicotine dissociation via C–C and C–N scission, yielding pyridinyl and methyl radicals, respectively; the former plays a role in nicotine degradation at low/mild temperatures, while the latter is only important at high temperatures due to its high activation energy. We also estimated nicotine lifetimes and branching ratios in order to identify health risks and understand the early steps of its degradation in different scenarios: high and mild temperatures, which are, respectively, the operating temperature of conventional and electronic cigarettes, and room temperature, which concerns indoor air quality exposure.

## 2 Methods

### 2.1 Electronic Structure Calculations and Conformational Search

The level of theory M06-2X/cc-pVTZ^31,32^ with an ultrafine grid was used to optimize and characterize the stationary points of the potential energy surface (PES) of the hydrogen abstraction reactions between nicotine and OH using the Gaussian16 software.^33^ In our conformational search, rotations within the rings of nicotine, hereafter pseudorotations, were explored manually; for each of the generated conformations, a further conformational search was performed by rotating the remaining *n* dihedrals by 90° using the MSTor 2013^34^ software, generating a total of 4*^n^* structures to explore for each case. The methyl group was not included in this search because it does not generate distinguishable structures. The optimized nicotine structures were used to generate possible saddle point structures by rotating the OH group by 60° to yield six feasible saddle point conformers per nicotine conformer and per abstraction site. The optimization of the saddle point conformers was performed with the QST3 method.^35^ Since the product species do not affect the rate constant calculation, a conformational search for the corresponding radical product species was not done. The M06-2X/aug-cc-pVQZ^31,32^ level of theory was used to refine the energy of all the optimized structures. Optimized geometries are provided in Section S6 of the Supporting Information.

The minimum energy conformer (global minimum) of each species was used to calculate the minimum energy path (MEP) with the Gaussrate17 software,^36^ and the rate constants were calculated with Polyrate 2016-2A.^37^ The MEPs were computed over the reaction coordinate range −1.36 to +1.36 bohr, with a stepsize of 0.1 bohr in isoinertial coordinates, employing a scaling mass factor of 1.0 amu and the Page–McIver method.^38^ A smaller stepsize of 0.0378 bohr was used for the reaction coordinate range −0.45 to +0.45 bohr in order to have a more accurate description of the variational transition state. Hessians along the MEPs were evaluated every third step, and normal modes were defined in Polyrate^37^ by using a set of curvilinear coordinates, which does not predict imaginary frequencies along the MEPs. A frequency scaling factor of 0.956^39^ was used for a more accurate description of torsional anharmonicity and the ground-state adiabatic potential energy curve; this curve, denoted as V_a_^G^(*s*), was used for the calculation of the tunneling transmission coefficients with the small-curvature tunneling approach (SCT)^40,41^ and is defined as
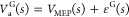
1where *V*_MEP_(*s*) is the classical potential energy defined with respect to that of the reactants, ε^G^(*s*) is the zero point energy (ZPE), and *s* is the reaction coordinate.

### 2.2 Multi-structural Torsional Variational Transition State Theory Calculations

The rate constants for the most kinetically favored pathways of the hydrogen abstraction reaction nicotine + OH → radical + H_2_O were computed using the multi-structural variational transition state theory^42^ with a coupled torsional potential^43,44^ and SCT tunneling corrections, hereafter labeled as *k*_MS – T(C)_^CVT/SCT^. First, we calculated rate constants using the global minimum conformers, the harmonic oscillator approximation, and the SCT method with the Polyrate 2016-2A code.^37^ Then, multi-structural torsional anharmonicity partition functions were obtained with MSTor 2013^34^ in order to include the effect of the multiple conformers or multi-structural anharmonicity as well as torsional anharmonicity. The calculations of the partition functions and rate constants with MSTor 2013 are explained in detail in Section S1 of the Supporting Information.

### 2.3 Variable Reaction Coordinate Calculations

The variable reaction coordinate formulation of the transition state theory (VRC-TST)^45,46^ is the most appropriate method to calculate rate constants of reactions without a saddle point, such as radical–radical association reactions.

We used this methodology to calculate the high-pressure limit (HPL) VRC-TST reverse rate constants of the C–N and C–C bond scission reactions of nicotine, that is, R_ass_-CN and R_ass_-CC; the calculated rate constants of the association reactions were used to calculate those for the dissociation reactions, R_diss_-CN and R_diss_-CC, by detailed balance. We first calculated the concentration equilibrium dissociation constants *K*_C_^diss^ (cm^–3^ molecule), which, together with the HPL VRC-TST association rate constant *k*_ass_^E, J – μVT^ (cm^3^ molecule^–1^ s^–1^), yields the corresponding HPL dissociation rate constant (s^–1^) as follows:
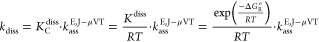
2where *K*^diss^ and Δ*G*_R_^o^ are the equilibrium constant and free energy of the dissociation reaction, respectively; the free energy was calculated with the thermodynamic functions derived from the multi-structural torsional anharmonicity partition functions of the reactants and products of each dissociation reaction.

Additional details for the application of the VRC-TST theory and the calculation of the *k*_ass_^E, J – μVT^ rate constants are provided in Section S2 of the Supporting Information.

### 2.4 Pressure-Dependent Rate Constant Calculations: SS-QRRK/MSC Approach

Pressure-dependent rate constants of the dissociation reactions were calculated with a recent modification implemented by us^47,48^ into the original system specific Rice–Ramsperger–Kessel theory (SS-QRRK) with the modified strong collision model (MSC) developed by Bao *et al*.,^49−51^ hereafter referred to as SS-QRRK/MSC. The SS-QRRK theory allows the inclusion of variational and multi-structural torsional anharmonicity effects, together with multidimensional tunneling contribution, in the low-pressure rate constants. The application of the SS-QRRK/MSC approach and the parameters used to estimate pressure effects are described in Section S3 of the Supporting Information.

## 3 Results and Discussion

### 3.1 Nicotine + OH Hydrogen Abstraction Reactions

#### 3.1.1 Stationary Points and Topology of the PES

**Figure 1:**
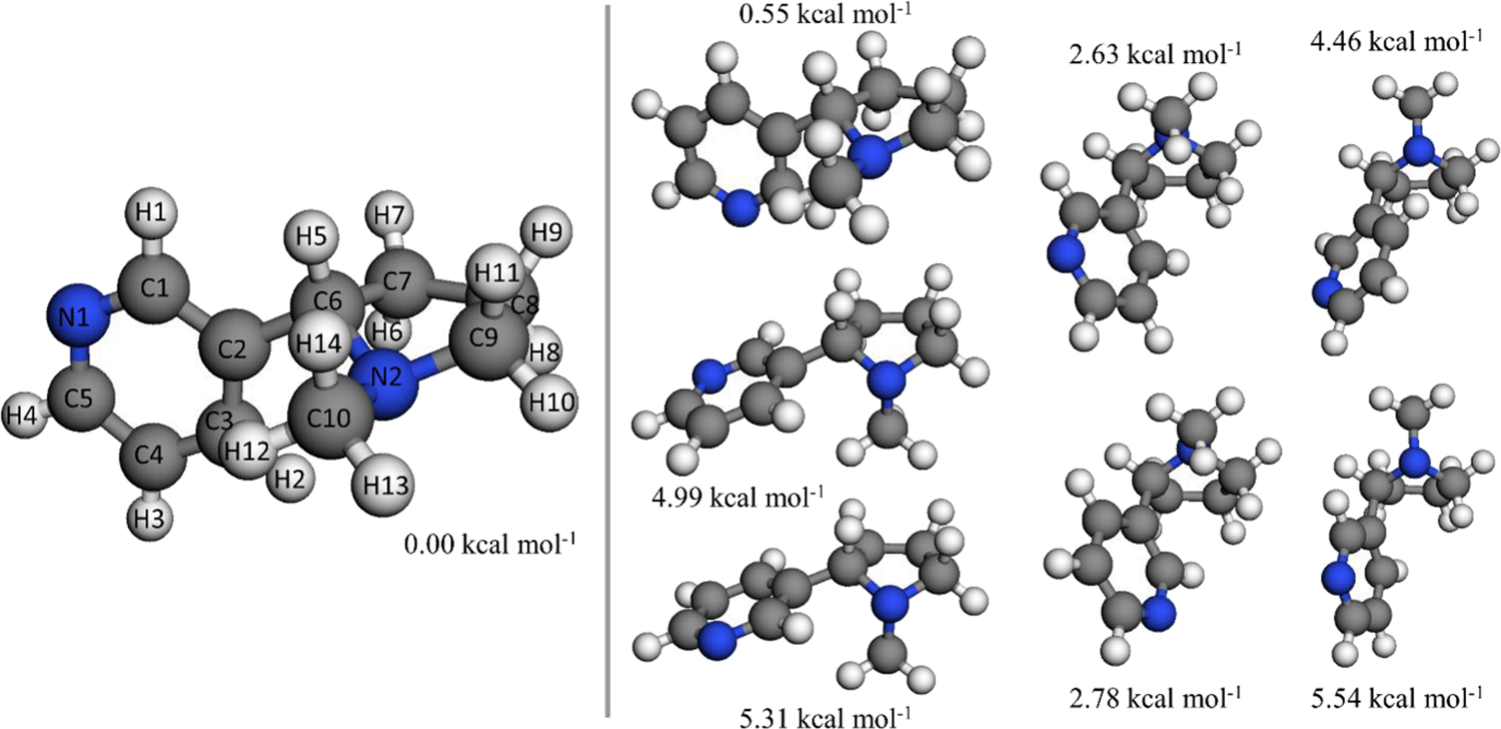
Optimized structures of the nicotine conformers at the M06-2X/cc-pVTZ level. Their potential energies at the M06-2X/aug-cc-pVQZ//M06-2X/cc-pVTZ level, defined with respect to the energy of the global minimum, are also displayed.

Figure 1 depicts the optimized geometries of the conformers of nicotine at the M06-2X/cc-pVTZ level and their potential energies defined with respect to that of the global minimum at the M06-2X/aug-cc-pVQZ//M06-2X/cc-pVTZ level. We considered the enantiomer *S* of nicotine since it is the one formed naturally;^52^ the rate constants of the reaction of the enantiomers *R* and *S* with nonchiral species are the same. We found eight distinguishable conformers for nicotine by rotating the dihedrals C1-C2-C6-N2 and N2-C6-C7-C8, which are the pseudorotation within the pyrrolidine ring responsible for its envelope conformation,^53,54^ and by changing the orientation of the methyl group bonded to the atom N2, which may display axial or equatorial orientation. All the displayed conformers have been reported in previous works.^53−58^

Previous experimental and theoretical works pointed out that at room temperature, around 99.9% of nicotine is found in the two lowest-energy conformations,^54^ with relative abundance values of 66.3% for the global minimum and 33.4%^57^ for the other one. However, at higher temperatures, such as those reached in cigarettes, the higher-energy conformers may become energetically accessible and thus a detailed multi-structural study is necessary in order to calculate accurate rate constants.

All the previous studies agree with our calculations about which of the different conformers of nicotine is the global minimum. In the global minimum structure, the two rings are the transversal one to the other, favoring an intramolecular hydrogen bond between the atoms H2 and N2.^53^ Additionally, we have observed that for those pairs of structures interconnected exclusively by rotating the dihedral angle C1-C2-C6-N2 by 180°, the conformer with the lowest energy is the one in which the distance between the nitrogen atoms is the longest, which agrees with the observations of Yoshida *et al.*(53)

Although nicotine can undergo the addition of OH to its aromatic ring, the hydrogen abstraction reaction has been found to be more kinetically favored.^2^ The abstraction of the hydrogen atom H5 (see Figure 1) yields a tertiary radical that can be further stabilized by resonant effects and by the adjacent electron-withdrawing N atom. Secondary radicals are obtained when the hydrogen atoms bonded to the atoms C7, C8, or C9 are abstracted, the former being stabilized to a larger extent by the electron-withdrawing N atom.^59^ Although abstraction from the methyl group would yield a less stable primary radical, it is also favored by the electron-withdrawing effect. The classical potential energy barriers for the abstraction of the 14 hydrogen atoms in the nicotine molecule by OH have been reported by Borduas *et al*.^2,19^ (Figure S2 in the Supporting Information). The abstraction sites located in the five-membered ring and the methyl group show negative barriers, which is in line with the stabilizing effect provided by the heteroatom.

**Figure 2:**
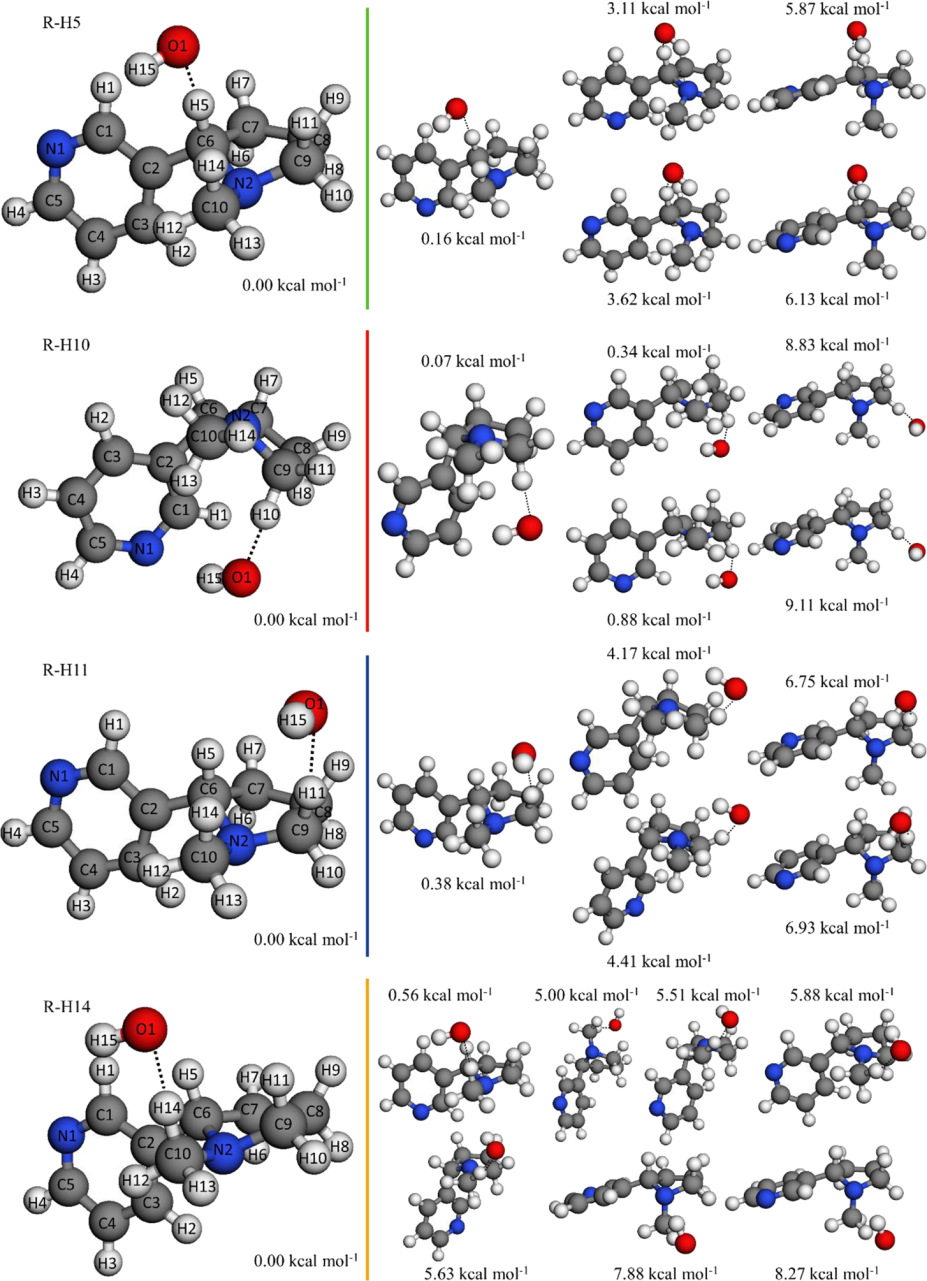
Optimized structures of the saddle point conformers at the M06-2X/cc-pVTZ level. Their potential energies at the M06-2X/aug-cc-pVQZ//M06-2X/cc-pVTZ level, defined with respect to the energy of the global minimum, are also displayed.

Addressing all the 14 hydrogen abstraction channels with the multi-structural torsional variational transition state theory is time-consuming; furthermore, several of these reactions are expected to have a marginal role in nicotine oxidation due to their larger barrier heights. Following the study by Borduas *et al*.,^2^ we approached the overall reaction between nicotine and OH by addressing the abstraction of the hydrogen atoms H5 and H11; the abstraction of H10 was likewise addressed since it yields the same radical product as H11. Since the energy barrier for the abstraction H14 is even lower than that of H10, we have also included the abstraction of H14 in our calculations. Therefore, we performed a robust kinetic study for the abstraction of the hydrogen atoms H5, H10, H11, and H14, hereafter referred to as reactions R-H5, R-H10, R-H11, and R-H14, respectively. We expect these four reactions to account for most of the overall rate constant for the reaction between nicotine and OH reported experimentally by Borduas *et al*.^2^ at 298 K, which will serve as a test to our theoretical approach. The results of the conformational search for the saddle points of those reactions are shown in Figure 2.

Figure S3 (Supporting Information) depicts the MEP computed using the M06-2X/cc-pVTZ level and scaled with the M06-2X/aug-cc-pVQZ energy using the global minimum conformers of the different species for the reactions R-H5, R-H10, R-H11, and R-H14. Single point energies of the products, intermediate complexes, and saddle points defined with respect to the energy of the reactants are presented in Table 1, including ZPE corrected values (adiabatic energies) with the 0.956^39^ scale factor. Our calculated barrier heights for the reactions R-H5, R-H10, R-H11, and R-H14 are 0.08, 0.38, 0.36, and 0.18 kcal mol^–1^ higher, respectively, than those reported by Borduas *et al*.,^2^ while the heat of reaction of R-H5 is 1.7 kcal mol^–1^ lower. These differences arise from the use of different levels of theory and conformers.

**Table 1:** Classical (Δ*E*) and Adiabatic (Δ*H*) Energies at the M06-2X/aug-cc-pVQZ//M06-2X/cc-pVTZ Level of the Stationary Points of the Reactions R-H5, R-H10, R-H11, and R-H14 Defined with Respect to the Energy of the Reactants

energy [kcal mol^–1^]	R-H5	R-H10	R-H11	R-H14
Δ*E*_RW_^a^	–4.17	–7.86	–3.21	–4.17
Δ*H*_RW_^a^	–3.11	–5.66	–2.41	–3.11
Δ*E*^‡b^	–3.12	–1.22	–2.64	–2.02
Δ*H*^‡b^	–2.83	–1.15	–2.79	–2.12
Δ*E*_PW_^c^	–42.92	–34.13	–33.51	–33.61
Δ*H*_PW_^c^	–41.52	–33.19	–32.37	–32.25
Δ*E*_RXN_^d^	–37.49	–27.34	–27.34	–25.75
Δ*H*_RXN_^d^	–37.61	–28.01	–28.01	–26.35

a RW: reactant well.

b ‡: saddle point.

c PW: product well.

d RXN: products.

The barrier heights reported in Table 1 were also calculated with the CCSD(T)/cc-pVTZ//M06-2X//cc-pVTZ level of theory in order to validate the performance of the M06-2X functional against the CCSD(T) *ab initio* method.^60^ For the reactions R-H5, R-H10, R-H11, and R-H14, we obtained the following classical barrier heights, respectively: −2.80, 0.33, −1.80, and −1.27 kcal mol^–1^. Both levels of theory predict that reaction R-H5 has the lowest barrier height followed by R-H11, R-H14, and R-H10, indicating that both would predict similar branching ratios. In addition, the barrier heights predicted by both levels of theory for the two most kinetically favored reactions, that is, R-H11 and R-H5, only differ by 0.84 and 0.32 kcal mol^–1^, respectively. Although the CCSD(T)/cc-pVTZ//M06-2X//cc-pVTZ level may predict different energy distributions for the multiple conformers and thus different global minimum conformers that may result in more submerged barrier heights, we conclude that the level of theory used in our kinetic study represents a reliable and cost-effective approach to the investigated reactions.

#### 3.1.2 Multi-structural Torsional Anharmonicity

**Figure 3:**
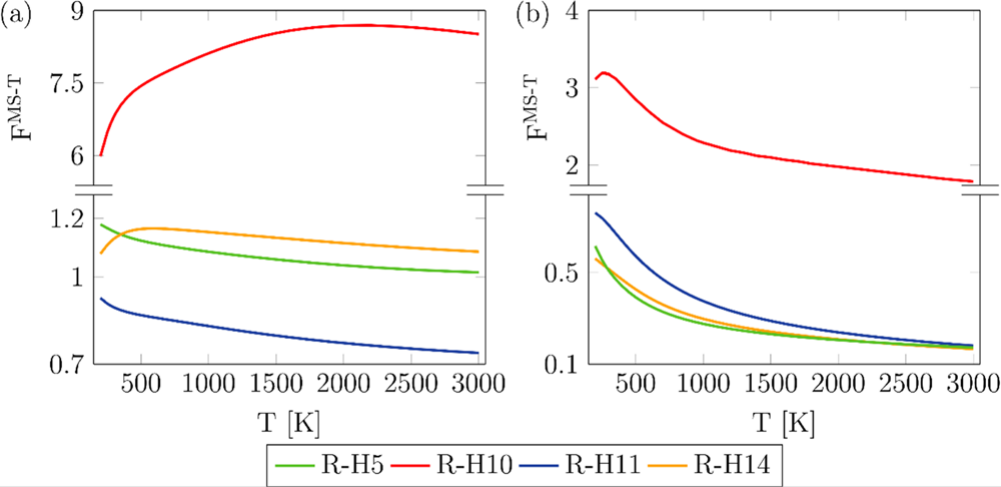
Effects of multi-structural anharmonicity (a) and multi-structural torsional anharmonicity (b) in reactions R-H5, R-H10, R-H11, and R-H114 as a function of temperature at the M06-2X/aug-cc-pVQZ//M06-2X/cc-pVTZ level.

The effects of the multiple conformers and torsional anharmonicity were included with the MSTor^34^ software. Table S1 (Supporting Information) shows the torsions considered for each species and the “Nearly Separable:Strongly Coupled” (NS:SC) scheme used to treat them. Figure 3a,b shows the multi-structural anharmonicity factor of each reaction calculated with the parameters defined by eq S6 and by eq S7 (Supporting Information), respectively; the former shows the effect of multi-structural anharmonicity, while the latter shows both, that is, multi-structural and torsional anharmonicity.

**Figure 4:**
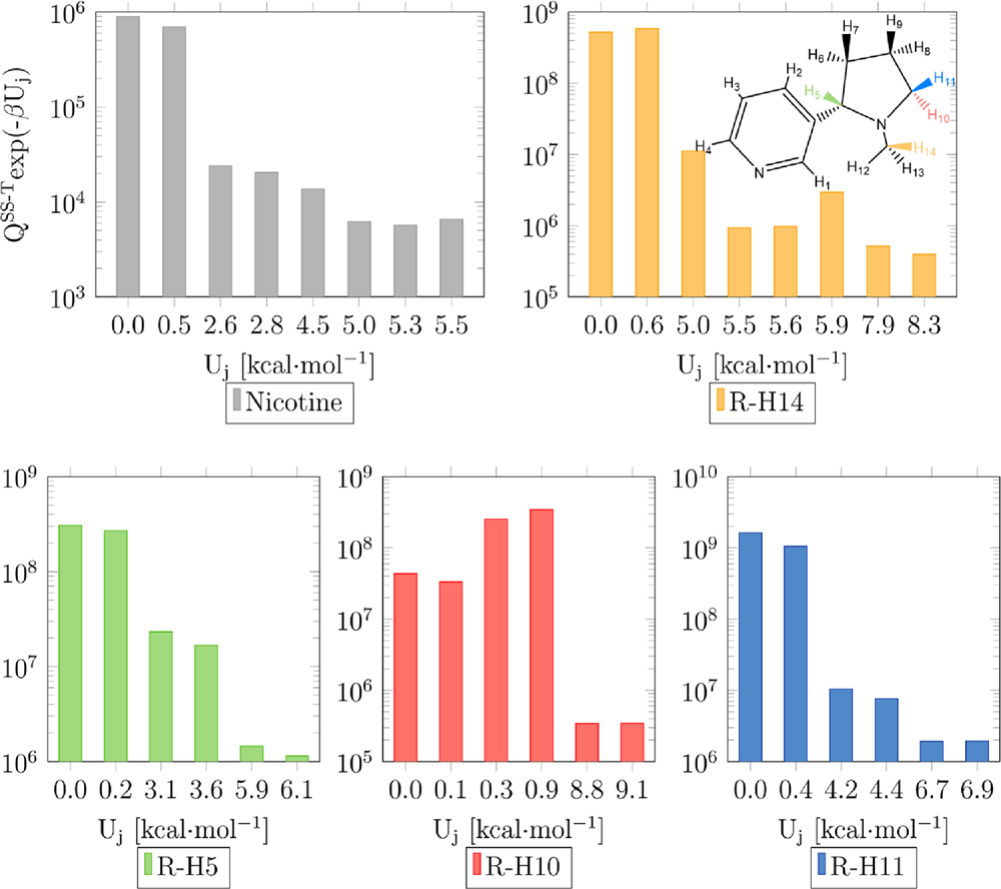
Contribution of the nicotine and saddle point conformers to their respective rovibrational multi-structural partition function at the M06-2X/aug-cc-pVQZ//M06-2X/cc-pVTZ level and 1500 K. The *x*-axis shows the potential energy distribution of the conformers defined with respect to that of the global minimum.

Multi-structural anharmonicity exerts a less pronounced effect in reactions R-H5, R-H11, and R-H14, while reaction R-H10 is significantly enhanced by this effect, especially at high temperatures. The saddle points of these reactions have a relatively similar number of distinguishable conformers, so these observations might be better explained by means of the contribution of those conformers to their corresponding rovibrational multi-structural partition function, which is addressed in Figure 4, including the nicotine species. Most of the conformers of the saddle points of reactions R-H5, R-H11, and R-H14 as well as those of nicotine lie at higher energies than the conformers of the saddle point of reaction R-H10; this is exemplified in Figure 4 at 1500 K, where it can be seen that four of the six conformers of the latter are within the more energetically accessible energy range of 0.0–1.0 kcal mol^–1^. As a result, the conformers of that saddle point play a more prominent role than the conformers of the other saddle points and nicotine species, justifying the positive effect of multi-structural anharmonicity in the case of reaction R-H10. This is not the case of reactions R-H5, R-H11, and R-H14, whose saddle point conformers show an equivalent role to those of nicotine, which result in a minor effect of multi-structural anharmonicity. Our findings demonstrate that even at low temperatures, the highest energy conformers of the saddle point of reaction R-H10 are determinant in the kinetics, indicating the need for a multi-structural treatment.

Interestingly, Figure 4 also shows that the global minimum of the saddle point of the reaction R-H10 does not play the main role in the kinetics, with two higher-energy conformers playing a larger role. This might be due to the hydrogen bond intermolecular interactions between the hydrogen atom of OH and the π electronic density of the pyridine ring and/or the two unpaired electrons of the N1 atom, which not only stabilize the global minimum but also add stiffness to the structure, decreasing its entropy and consequently increasing the free energy. The geometry of the two lowest energy conformers of this saddle point favors these hydrogen bond intermolecular interactions by positioning the OH and pyridine ring perpendicularly; this can be seen in Figure 2, with the other conformers not showing such an orientation. Strong OH···π hydrogen bonds and entropy effects have been reported to be relevant in other reactive systems.^61,62^

Figure 3b illustrates the effect of both multi-structural and torsional anharmonicity on the four reactions, indicating that the missing torsional anharmonicity would also lead to large errors.

#### 3.1.3 Rate Constants and Branching Ratios

**Figure 5:**
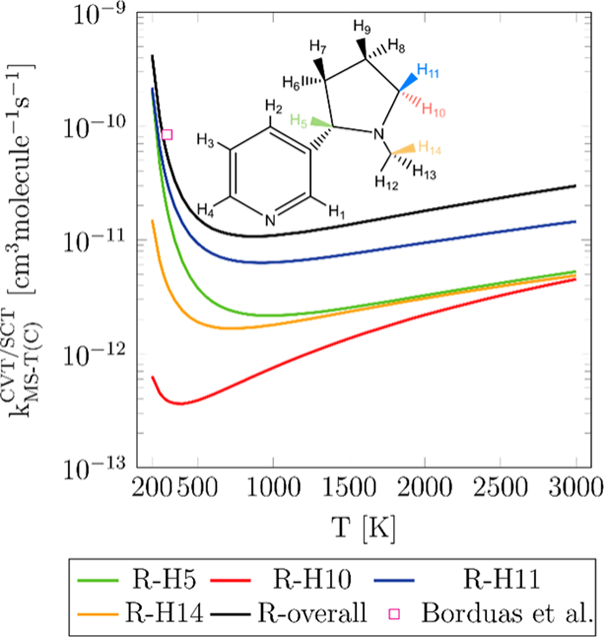
Rate constants as a function of temperature for reactions R-H5, R-H10, R-H11, and R-H14 computed with the multi-structural torsional variational transition state theory at the M06-2X/aug-cc-pVQZ//M06-2X/cc-pVTZ level. The overall rate constants, calculated as the sum of the site-specific rate constants, and the experimental value by Borduas *et al*.^2^ are also shown.

Figure 5 shows the rate constants as a function of temperature for the abstraction of the hydrogen atoms H5, H10, H11, and H14 by OH from nicotine as well as the overall rate constants as the sum of them. In opposition to what the potential energy barriers suggest, the rate constants for reaction R-H11 are faster than those for R-H5. Interestingly, the reaction R-H14 competes to a similar extent with reaction R-H5 at temperatures higher than 1200 K, despite the fact of the latter having a lower potential energy barrier. This indicates that there might be other effects, such as entropy and free energy, which have to be considered for this comparison.

**Figure 6:**
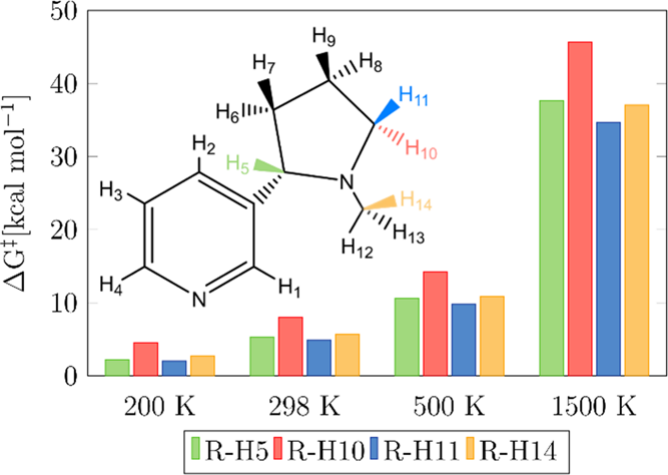
Gibbs free-energy barrier as a function of temperature for reactions R-H5, R-H10, R-H11, and R-H14 computed at the M06-2X/aug-cc-pVQZ//M06-2X/cc-pVTZ level.

Figure 6 depicts the free-energy barriers of each reaction at several temperatures. The free-energy barrier for R-H11 is always the lowest among the assessed reactions, explaining its prominence. At temperatures lower than or equal to 500 K, the free-energy barrier for the reaction R-H5 is slightly lower than that for R-H14, explaining why the former reaction is faster than the latter in that temperature range. Nonetheless, at higher temperatures, the free-energy barrier for R-H14 becomes similar to that for R-H5, explaining the trend observed in Figure 5. Reaction R-H10, with the highest free-energy barrier at all the considered temperatures, represents a minor contribution to the overall rate constant, although it is significantly enhanced as temperature increases by the effect of multi-structural torsional anharmonicity.

The different temperature trends at low and high temperatures of the calculated rate constants are due to the submerged barrier of the four reactions, which induces a change in the sign of the activation energy^63^ and explains the observed minima in the rate constants (located at 873 K for the overall reaction). This non-Arrhenius behavior has implications in the degradation mechanism and lifetime of nicotine, as will be discussed later. Tunneling is not playing any role in these reactions and thus is not involved in the enhancement of the rate constants at low temperatures.

The overall rate constants for the hydrogen abstraction are also presented in Figure 5 and are compared with the only available experimental value reported by Borduas *et al*.^2^ at 298 K, exhibiting excellent agreement with a relative error of −31.3%. Our calculated rate constants and barrier heights indicate that the abstraction of the remaining hydrogen atoms, that is, H6, H7, H8, H9, H12, and H13, can be considered as irrelevant; this conclusion is supported by two factors: first, our calculated rate constant at 298 K based on the reactions R-H5, R-H10, R-H11, and R-H14 reproduces very well the experimental value, and second, the fact that the reaction R-H10, with the highest potential and free-energy barriers among those included in our kinetic study, only shows a minor contribution to the total rate constant indicates that those missing reactions, with larger potential energy barriers, are even less relevant and can be safely disregarded in the oxidation mechanism of nicotine. We attribute the small discrepancy observed between our calculated rate constant and the only available experimental value at 298 K to the errors inherent to any kinetic study, such as errors in the calculated barrier heights.

All the rate constant values plotted in Figure 5 are provided in the Supporting Information and were fitted to the modified Arrhenius expression. The fitting parameters are presented in Table S3 in the Supporting Information.

**Figure 7:**
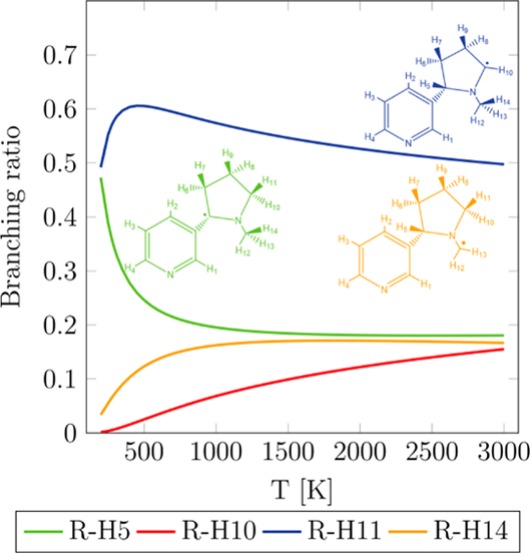
Branching ratios of reactions R-H5, R-H10, R-H11, and R-H14 as a function of temperature obtained with the calculated rate constants shown in Figure 5. For a given reaction R-Hj, the branching ratio is calculated as *k*_R-Hj_/*k*_R-overall_.

The branching ratios for the reaction of nicotine with OH are difficult to obtain experimentally; hence, our theoretical study represents a useful tool to have better insights into the oxidation process of nicotine. This information is displayed in Figure 7, where it can be seen that the reaction R-H11 is the prominent one. Indeed, within the temperature range 600–1200 K, which includes typical operating temperatures for the different smoking devices and regular cigarettes, the contribution of the reaction R-H11 varies between 56 and 60%. Interestingly, it is more important than the reaction R-H5, which yields a much more stable tertiary radical (Table 1); we attribute these findings to the entropy effects that make the reaction R-H5 go over a higher free-energy barrier. The radicals yielded by reactions R-H10 and R-H14 will be barely formed at temperatures below 600 K, becoming a bit more prominent as temperature rises due to the multi-structural anharmonicity contribution, which is especially pronounced in reaction R-H10. The secondary nicotine radical generated by reactions R-H10 and R-H11 will show the highest yield when nicotine reacts with OH, representing at least 50% of the total radical pool.

**Figure 8:**
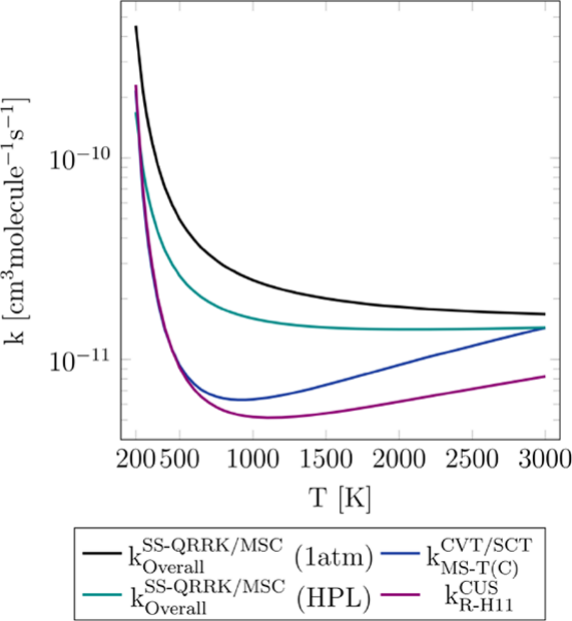
Comparison of the rate constants predicted for reaction R-H11 by the CUS model (*k*_R – H11_^CUS^, high-pressure limit), the SS-QRRK/MSC approach (*k*_Overall_^SS – QRRK/MSC^, high-pressure limit and 1 atm), and the approach that assumes a negligible effect of the reactant complex (*k*_MS – T(C)_^CVT/SCT^, high-pressure limit).

The effect that the reactant complex of reaction R-H11, which is the most prominent one, has in the calculated rate constants was estimated with the canonical unified statistical model (CUS)^64^ and the SS-QRRK/MSC approach.^47,48^ The former considers the formation of the reactant complex from the reactants, that is, nicotine + OH → reactant complex, whose rate constants were calculated with the VRC-TST formulation and are labeled as *k*_ass, Complex_^E, J – μVT^; the CUS rate constants for the overall process are then estimated as follows:
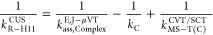
3where *k*_C_ is a rate constant calculated for a dividing surface that is located at the free energy minimum that corresponds to the reactant complex and *k*_MS – T(C)_^CVT/SCT^ is that calculated for reaction R-H11 assuming a negligible effect of the complex (Figure 5). Since *k*_ass, Complex_^E, J – μVT^ was calculated in the high-pressure limit, our *k*_R – H11_^CUS^ values can be only used to check the effect of the reactant complex in that pressure regime; however, those obtained with the SS-QRRK/MSC approach, which are labeled as *k*_Overall_^SS – QRRK/MSC^ and implicitly consider the effect of the reactant complex by assuming the overall process nicotine + OH → reactant complex → products, were also derived at low pressures. Details regarding the calculation of *k*_ass, Complex_^E, J – μVT^ and *k*_Overall_^SS – QRRK/MSC^ are provided in Sections S2 and S3, respectively, of the Supporting Information. In Figure 8, we show the rate constant values predicted by the different approaches that we are comparing for reaction R-H11 (values are provided in Table S4 of the Supporting Information).

It can be seen that the different approaches predict very similar results for the high-pressure limit and also that pressure effects are not very pronounced; the largest difference is observed between the *k*_Overall_^SS – QRRK/MSC^ and *k*_R – H11_^CUS^ approaches at 650 K, the former being 3.3 times larger than the latter. Although the SS-QRRK/MSC approach can be in principle expected to yield more accurate rate constants than the others as a result of the implicit consideration of the complex, it should be also noted that this approach applies the steady-state approximation to the chemical activation mechanism; the concentration of the complex may however deviate from the steady-state condition, introducing some uncertainty in this approach. It is therefore complicated to determine which model describes with more fidelity the dynamic behavior of the reactive system, i.e., the complex-mediated or direct mechanism; we believe that it may depend on the conditions, mainly temperature as pressure does not exert an important effect.

To shed light into this interesting question, we refer to the work by Monge-Palacios *et al*.^65^ for the reaction NH_3_ + OH → NH_2_ + H_2_O, whose reaction mechanism was elucidated at the atomic level with a detailed molecular dynamics study; similar to the hydrogen abstraction reactions that we are investigating, this reaction shows a reactant complex whose potential energy barrier separating it from the saddle point is also comparable. In that work, the authors found that at 298 K, only 2% of the reactive encounters formed the reactant complex and that percentage becomes lower as temperature is increased. Therefore, we believe that the assumption of a direct and nonpressure-dependent mechanism, instead of the complex-mediated mechanism described with the SS-QRRK/MSC approach, may represent a better description of the hydrogen abstraction reactions between nicotine and the hydroxyl radical. This conclusion is also supported by the better agreement between the experimental rate constant reported by Borduas *et al.*(2) at 298 K and atmospheric pressure and our *k*_MS – T(C)_^CVT/SCT^ value.

We did not find any experimental study reporting final or intermediate oxidation products of nicotine in the gas phase to validate our findings; however, Passananti *et al.*(66) reported with HPLC-MS experiments the chemical structures of the intermediates formed in aqueous nicotine oxidation by OH, which were considered to be derived from the radicals yielded by the reactions R-H11 and R-H5 via reactions such as OH and O_2_ addition.

### 3.2 Nicotine Dissociation Reactions

#### 3.2.1 Rate Constants

**Figure 9:**
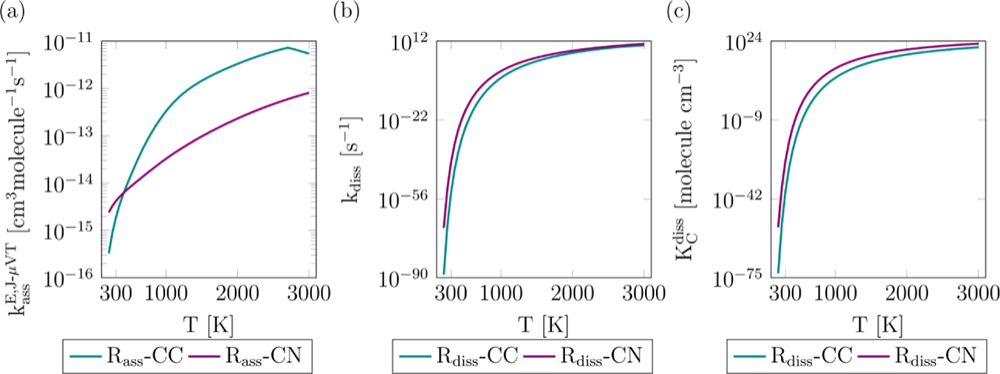
VRC-E,J-μVT rate constants of the radical–radical association reactions that form nicotine calculated at the MN15-L/cc-pVTZ level (a). Rate constants of the R_diss_-CN and R_diss_-CC nicotine dissociation reactions (b). Concentration equilibrium constants for the R_diss_-CN and R_diss_-CC nicotine dissociation reactions (c).

The rate constants for the radical–radical association reactions R_ass_-CN and R_ass_-CC yielding nicotine that were obtained with the VRC-E,J-μVT theory in the high-pressure limit are plotted in Figure 9, together with the corresponding nicotine dissociation rate constants R_diss_-CN and R_diss_-CC that were obtained by detailed balance and the concentration equilibrium constants for the dissociation reactions *K*_C_^diss^. Our calculations indicate that the dissociation of the C–N bond is more likely in the whole temperature range, especially at intermediate and low temperatures. Pressure effects were found to be unimportant, and similar rate constants were obtained even at pressures as low as 0.1 bar. From our kinetic study, we conclude that the methyl and 3-(pyrrodin-2-yl)pyridinyl radicals will be much more likely formed than pyridinyl and 1-methylpyrrolidinyl radicals in nicotine decomposition; this is in agreement with the reaction energies that we calculated with the MN15-L/cc-pVTZ level for reactions R_diss_-CN and R_diss_-CC, with values of 82.39 and 96.94 kcal mol^–1^, respectively. The optimal dividing surfaces were located at *r*_C-N_ = 2.6 Å and *r*_C-C_ = 5.0 Å, indicating a much earlier transition state for the reaction R_ass_-CC.

These rate constants were fitted to the modified Arrhenius equation, whose fitting parameters are shown in Table S7 in the Supporting Information. The values of the rate and equilibrium constants shown in Figure 9 are provided in the Supporting Information.

### 3.3 Degradation and Lifetime of Nicotine

OH is one of the most important atmospheric oxidizers in indoor and outdoor environments, and the C–N and C–C bond dissociation reactions tackled in this work are the most likely ones in thermal decomposition of nicotine. Therefore, our calculated rate constants can serve to unravel the early chemistry of nicotine degradation in different environments.

**Figure 10:**
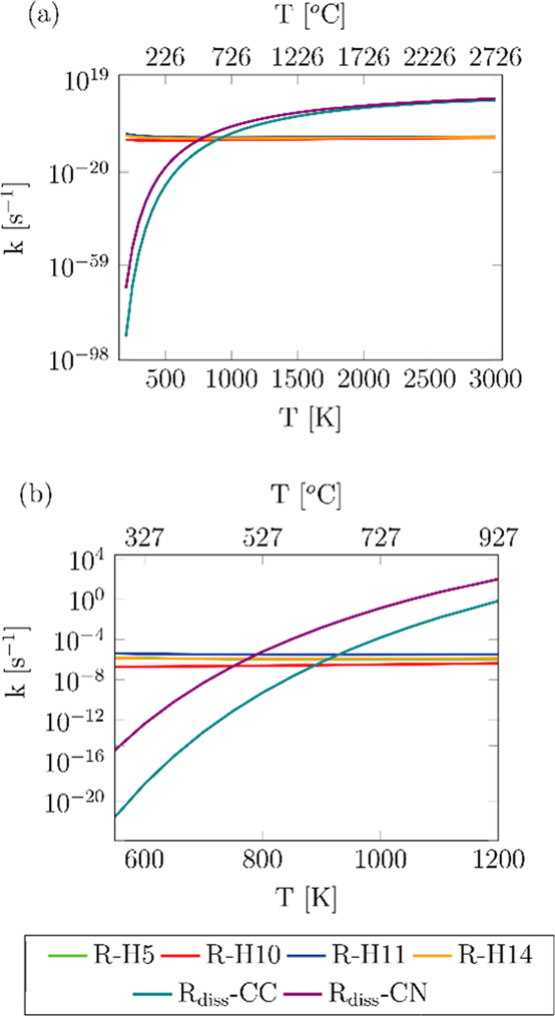
Comparison between the calculated pseudo-first-order rate constants of the investigated hydrogen abstraction reactions and nicotine dissociation rate constants using an indoor concentration of OH of 5 × 10^5^ molecules cm^–3^ (a). Same results for a narrower temperature interval (b).

For a more appropriate comparison of the different bimolecular and unimolecular reactions considered in this work, the rate constants of the hydrogen abstraction reactions were converted into pseudo-first-order reactions by multiplying them by the concentration of OH; for this purpose, we used an indoor concentration of OH radicals of 5 × 10^5^ molecules cm^–3^. This comparison is shown in Figure 10; it should be noted that when using higher concentrations of OH, such as 2 × 10^6^ molecules cm^–3^, which is typical in outdoor environments, very similar results were derived.

We conclude that the nondegraded nicotine released in the smoke of cigarettes will not undergo neither C–N nor C–C dissociation in indoor and outdoor environments. Instead, it will more likely react quickly with OH to generate the corresponding radical, which is mainly that from reactions R-H11 and R-H14, as well as with other species present in indoor and outdoor environments, which are not the scope of the present work; these radicals will continue degradation toward other harmful species such as formamide and isocyanic acid,^2^ and thus we can consider the formation of the harmful pyridinyl radical via C–C scission,^20^ and eventually pyridine, as highly unlikely.

However, the scenario for the nicotine that is degraded in the cigarette while smoking is different, especially in conventional cigarettes whose operating temperatures are much higher than those of electronic cigarettes and vaporizers. In the former case, with temperatures of even 1200 K, dissociation reactions are more prominent than hydrogen abstraction by OH, and the former may become competitive in nicotine degradation; therefore, pyridinyl radicals can be expected in the smoke radical pool, although to a lower extent than 3-(pyrrodin-2-yl)pyridinyl and methyl radicals. Electronic cigarettes and vaporizers may prevent the formation of the radicals resulting from nicotine dissociation due to their lower operating temperatures of around 600 K, leading to a different aerosol matrix; however, it would be interesting to investigate the fate of the radicals formed by the reaction of nicotine with OH, which seems to control the toxicity of the aerosol released by these smoking devices.

The rate constants reported in Figure 10 were used to estimate the lifetime of nicotine. For the nicotine that is not degraded in the cigarette and instead is released to the environment, lifetime was approached by only considering the hydrogen abstraction reactions since dissociation reactions are extremely slow at low/intermediate temperatures:
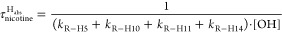
4where the bimolecular rate constants of the hydrogen abstraction reactions were converted into pseudo-first-order rate constants, as was previously discussed. For the nicotine that is subjected to higher temperatures in the cigarette, which is more prone to dissociate, lifetime was estimated as
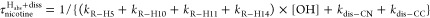
5

**Figure 11:**
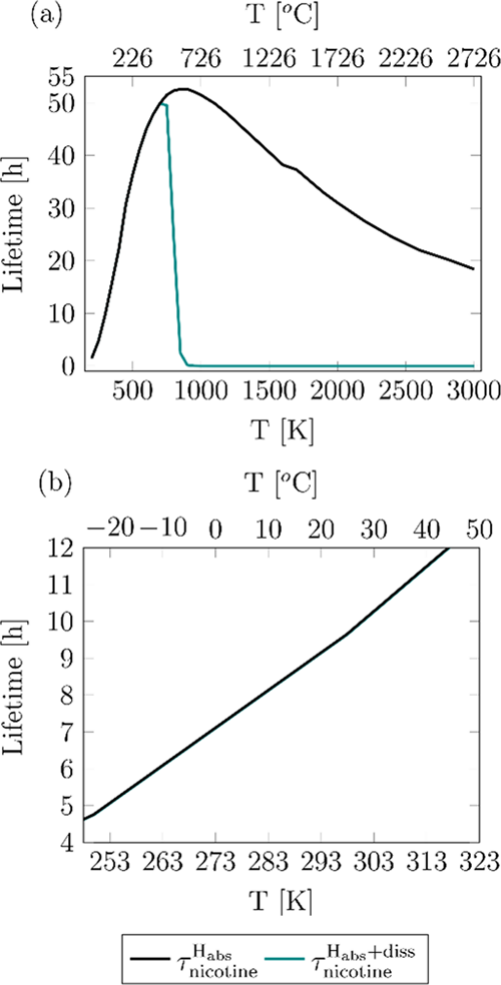
Lifetimes as a function of temperature calculated for the nicotine that is released to the environment (τ_nicotine_^H_abs_^) and for the nicotine that is degraded in cigarettes at higher temperatures (τ_nicotine_^H_abs_ + diss^) (a). Lifetimes in a narrower temperature range (b).

The calculated lifetimes are plotted in Figure 11, where it can be seen that both approaches predict similar results at temperatures below 750 K when the dissociation mechanism is negligible. Interestingly, this temperature defines two different trends in nicotine lifetimes and degradation, resulting in two different scenarios. The lifetime of the nicotine that is exposed to temperatures beyond 750 K in conventional cigarettes is rapidly reduced when temperature is increased; it has been reported that in burning cigarettes, there might be short-lived hot spots with temperatures as high as 1400 K,^67^ which would be critical for the yield of radicals from C–C and C–N nicotine dissociation such as pyridinyl radicals, with important health implications. However, nicotine in low-temperature operating smoking devices as well as the nicotine released to the environment is degraded at a slower rate and therefore lasts longer, as temperature increases up to 750 K; this different trend results from the different nicotine degradation mechanism that takes place in this low-temperature regime, that is, hydrogen abstraction by OH, which implies a longer exposure to nicotine and eventually to the radicals formed after its reaction with OH not only for smokers but also for second- and third-hand smokers. The maximum in the τ_nicotine_^H_abs_^ curve and thus the change in its temperature dependence are observed at approximately the same temperature as the minimum in the rate constants plotted in Figure 5 for the overall hydrogen abstraction reaction, highlighting the implications of the non-Arrhenius behavior of those rate constants.

It is important to highlight that our lifetime analysis is based on the reactions that we have investigated in the present work. Although the OH radical is one of the most important species involved in oxidative processes and the two unimolecular dissociation reactions that we considered have been previously reported by other authors,^7,20^ our analysis would be further benefitted by the consideration of reactions that involve other important species that are present in indoor and outdoor environments as well as in tobacco smoke.

## 4 Conclusions

We performed a robust theoretical kinetic study and found out that different initiation mechanisms can take place in the oxidation and pyrolysis of nicotine, resulting in a complex degradation process. At temperatures below 800 K, hydrogen abstraction reactions by OH radicals are expected to play an important role in the oxidation process due to the high activation energy of the bond dissociation reactions, which instead are more likely to control nicotine degradation at higher temperatures. In addition, we observed that hydrogen abstraction reactions by OH radicals are enhanced as temperature decreases below 873 K due to their non-Arrhenius kinetics. As a consequence, our calculated lifetimes for nicotine in indoor environments are longer as temperature is reduced, resulting in longer exposure of room occupants to nicotine. The calculated rate constants and nicotine lifetimes, which include multi-structural torsional anharmonicity effects, are validated by the only experimental value available at 298 K; both calculated and experimental rate constants indicate a fast oxidation mechanism with values of, respectively, 5.76 × 10^–11^ and 8.38 × 10^–11^ cm^3^ molecule^–1^ s^–1^.

Our calculated rate constants suggest a different nicotine degradation mechanism in conventional cigarettes and heat-not-burn devices. The operating temperatures of the former are around 900 °C (1173 K), which promotes the dissociation of nicotine and yields harmful pyridinyl radicals that are inhaled by smokers and second- and third-hand smokers; the latter, whose operating temperatures are below 600 K (327 °C), will likely degrade nicotine via other reactions such as hydrogen abstraction by OH radicals, yielding different nicotine radicals that may be precursors of other harmful species. Although the smoke of these low-temperature smoking devices such as IQOS may not be as harmful as that of conventional cigarettes due to its lower content in toxic chemicals, their operating temperatures may not be the optimal ones as they are actually enhancing nicotine oxidation by entering the region below 873 K in which hydrogen abstraction reactions from nicotine by OH radicals rapidly increase as temperature is reduced, promoting the formation of harmful species in contrast to what is claimed by manufacturers. We conclude that heat-not-burn devices may not be as harmless as it is believed and also that operating temperatures around 873 K, when hydrogen abstraction by OH radicals shows the slowest rate, may be more effective in hindering the formation of harmful species.

Although our work represents a good starting point to understand the early chemistry of nicotine degradation in different environments, additional theoretical and experimental studies are necessary to fully unravel this process. Considering other important species that are present in those environments and may react with nicotine as well as alternative nicotine dissociation pathways would be appropriate to accomplish that goal.

## Supporting Information Available

Details about the calculations performed and the methods used as well as additional results (PDF)

## References

[ref1] World Health Organization. Tobacco; https://www.who.int/news-room/fact-sheets/detail/tobacco (accessed Aug 11, 2020).

[ref2] Borduas N.; Murphy J. G.; Wang C.; Da Silva G.; Abbatt J. P. D. Gas Phase Oxidation of Nicotine by OH Radicals: Kinetics, Mechanisms, and Formation of HNCO. Environ. Sci. Technol. Lett. 2016, 3, 327–331. 10.1021/acs.estlett.6b00231.

[ref3] Valavanidis A.; Vlachogianni T.; Fiotakis K. Tobacco Smoke: Involvement of Reactive Oxygen Species and Stable Free Radicals in Mechanisms of Oxidative Damage, Carcinogenesis and Synergistic Effects with Other Respirable Particles. Int. J. Environ. Res. Public Health 2009, 6, 445–462. 10.3390/ijerph6020445. 19440393 PMC2672368

[ref4] Ilies B. D.; Moosakutty S. P.; Kharbatia N. M.; Sarathy S. M. Expression of concern: Identification of Volatile Constituents Released from IQOS Heat-Not-Burn Tobacco HeatSticks Using a Direct Sampling Method. Tob. Control 2020, 055521 10.1136/tobaccocontrol-2019-055521. PMC12171520 32457207

[ref5] Newman M. B.; Arendash G. W.; Shytle R. D.; Bickford P. C.; Tighe T.; Sanberg P. R. Nicotine’s Oxidative and Antioxidant Properties in CNS. Life Sci. 2002, 71, 2807–2820. 10.1016/S0024-3205(02)02135-5. 12377264

[ref6] U.S. Department of Health and Human Services. Smoking Cessation. A Report of the Surgeon General; Department of Health and Human Services, Centers for Disease Control and Prevention, National Center for Chronic Disease Prevention and Health Promotion, Office on Smoking and Health: Atlanta, GA, 2020.

[ref7] Kurgat C.; Kibet J.; Cheplogoi P. Molecular Modeling of Major Tobacco Alkaloids in Mainstream Cigarette Smoke. Chem. Cent. J. 2016, 10, 43 10.1186/s13065-016-0189-5. 27429644 PMC4947291

[ref8] Destaillats H.; Singer B. C.; Lee S. K.; Gundel L. A. Effect of Ozone on Nicotine Desorption from Model Surfaces: Evidence for Heterogeneous Chemistry. Environ. Sci. Technol. 2006, 40, 1799–1805. 10.1021/es050914r. 16570600

[ref9] Singer B. C.; Revzan K. L.; Hotchi T.; Hodgson A. T.; Brown N. J. Sorption of Organic Gases in a Furnished Room. Atmos. Environ. 2004, 38, 2483–2494. 10.1016/j.atmosenv.2004.02.003.

[ref10] Singer B. C.; Hodgson A. T.; Nazaroff W. W. Gas-Phase Organics in Environmental Tobacco Smoke: 2. Exposure-Relevant Emission Factors and Indirect Exposures from Habitual Smoking. Atmos. Environ. 2003, 37, 5551–5561. 10.1016/j.atmosenv.2003.07.015.

[ref11] Bush D.; Goniewicz M. L. A Pilot Study on Nicotine Residues in Houses of Electronic Cigarette Users, Tobacco Smokers, and Non-Users of Nicotine-Containing Products. Int. J. Drug Policy 2015, 26, 609–611. 10.1016/j.drugpo.2015.03.003. 25869751 PMC4457620

[ref12] Wang C.; Collins D. B.; Hems R. F.; Borduas N.; Antiñolo M.; Abbatt J. P. D. Exploring Conditions for Ultrafine Particle Formation from Oxidation of Cigarette Smoke in Indoor Environments. Environ. Sci. Technol. 2018, 52, 4623–4631. 10.1021/acs.est.7b06608. 29601184

[ref13] Sleiman M.; Gundel L. A.; Pankow J. F.; Jacob P.; Singer B. C.; Destaillats H. Formation of Carcinogens Indoors by Surface-Mediated Reactions of Nicotine with Nitrous Acid, Leading to Potential Thirdhand Smoke Hazards. Proc. Natl. Acad. Sci. U. S. A. 2010, 107, 6576–6581. 10.1073/pnas.0912820107. 20142504 PMC2872399

[ref14] Wells J. R.; Schoemaecker C.; Carslaw N.; Waring M. S.; Ham J. E.; Nelissen I.; Wolkoff P. Reactive Indoor Air Chemistry and health—A Workshop Summary. Int. J. Hyg. Environ. Health 2017, 220, 1222–1229. 10.1016/j.ijheh.2017.09.009. 28964679 PMC6388628

[ref15] Holland F.; Hofzumahaus A.; Schäfer J.; Kraus A.; Pätz H.-W. Measurements of OH and HO_2_ Radical Concentrations and Photolysis Frequencies during BERLIOZ. J. Geophys. Res. Atmos. 2003, 108, PHO 2-1–PHO 2-23. 10.1029/2001JD001393.

[ref16] Alvarez E. G.; Amedro D.; Afif C.; Gligorovski S.; Schoemaecker C.; Fittschen C.; Doussin J.-F.; Wortham H. Unexpectedly High Indoor Hydroxyl Radical Concentrations Associated with Nitrous Acid. Proc. Natl. Acad. Sci. U. S. A. 2013, 110, 13294–13299. 10.1073/pnas.1308310110. 23898188 PMC3746940

[ref17] Jacob D. J. Introduction to Atmospheric Chemistry; Princeton University Press: NJ, 1999.

[ref18] Carslaw N.; Fletcher L.; Heard D.; Ingham T.; Walker H. Significant OH Production under Surface Cleaning and Air Cleaning Conditions: Impact on Indoor Air Quality. Indoor Air 2017, 27, 1091–1100. 10.1111/ina.12394. 28493625

[ref19] Borduas N. The Atmospheric Fate of Organic Nitrogen Compounds. Ph.D. Dissertation, University of Toronto: Toronto, 2015.

[ref20] Kibet J.; Kurgat C.; Limo S.; Rono N.; Bosire J. Kinetic Modeling of Nicotine in Mainstream Cigarette Smoking. Chem. Cent. J. 2016, 10, 60 10.1186/s13065-016-0206-8. 27790285 PMC5062895

[ref21] Kehrer J. P.; Mossman B. T.; Sevanian A.; Trush M. A.; Smith M. T. Free Radical Mechanisms in Chemical Pathogenesis: Summary of the Symposium Presented at the 1988 Annual Meeting of the Society of Toxicology. Toxicol. Appl. Pharmacol. 1988, 95, 349–362. 10.1016/0041-008X(88)90354-7. 3188006

[ref22] Dellinger B.; Pryor W. A.; Cueto R.; Squadrito G. L.; Hegde V.; Deutsch W. A. Role of Free Radicals in the Toxicity of Airborne Fine Particulate Matter. Chem. Res. Toxicol. 2001, 14, 1371–1377. 10.1021/tx010050x. 11599928

[ref23] Dellinger B.; Pryor W. A.; Cueto B.; Squadrito G. L.; Deutsch W. A. The Role of Combustion-Generated Radicals in the Toxicity of PM2.5. Proc. Combust. Inst. 2000, 28, 2675–2681. 10.1016/S0082-0784(00)80687-6.

[ref24] Borgerding M.; Klus H. Analysis of Complex Mixtures – Cigarette Smoke. Exp. Toxicol. Pathol. 2005, 57, 43–73. 10.1016/j.etp.2005.05.010. 16092717

[ref25] Baker R. R. Temperature Distribution inside a Burning Cigarette. Nature 1974, 247, 405–406. 10.1038/247405a0.

[ref26] Smith M. R.; Clark B.; Lüdicke F.; Schaller J.-P.; Vanscheeuwijck P.; Hoeng J.; Peitsch M. C. Evaluation of the Tobacco Heating System 2.2. Part 1: Description of the System and the Scientific Assessment Program. Regul. Toxicol. Pharmacol. 2016, 81, S17–S26. 10.1016/j.yrtph.2016.07.006. 27450400

[ref27] Ibañez M. P.; Martin D.; Gonzálvez A. G.; Telle H. H.; Ureña Á. G. A Comparative Study of Non-Volatile Compounds Present in 3R4F Cigarettes and iQOS Heatsticks Utilizing GC-MS. Am. J. Anal. Chem. 2019, 10, 76–85. 10.4236/ajac.2019.103007.

[ref28] Bentley M. C.; Almstetter M.; Arndt D.; Knorr A.; Martin E.; Pospisil P.; Maeder S. Comprehensive Chemical Characterization of the Aerosol Generated by a Heated Tobacco Product by Untargeted Screening. Anal. Bioanal. Chem. 2020, 412, 2675–2685. 10.1007/s00216-020-02502-1. 32072212 PMC7136312

[ref29] Bitzer Z. T.; Goel R.; Trushin N.; Muscat J.; Richie J. P. Jr. Free Radical Production and Characterization of Heat-Not-Burn Cigarettes in Comparison to Conventional and Electronic Cigarettes. Chem. Res. Toxicol. 2020, 33, 1882–1887. 10.1021/acs.chemrestox.0c00088. 32432464 PMC9328730

[ref30] Shein M.; Jeschke G. Comparison of Free Radical Levels in the Aerosol from Conventional Cigarettes, Electronic Cigarettes, and Heat-Not-Burn Tobacco Products. Chem. Res. Toxicol. 2019, 32, 1289–1298. 10.1021/acs.chemrestox.9b00085. 30932480 PMC6584902

[ref31] Zhao Y.; Truhlar D. G. The M06 Suite of Density Functionals for Main Group Thermochemistry, Thermochemical Kinetics, Noncovalent Interactions, Excited States, and Transition Elements: Two New Functionals and Systematic Testing of Four M06-Class Functionals and 12 Other Function. Theor. Chem. Acc. 2008, 120, 215–241. 10.1007/s00214-007-0310-x.

[ref32] Dunning T. H. Jr. Gaussian Basis Sets for Use in Correlated Molecular Calculations. I. The Atoms Boron through Neon and Hydrogen. J. Chem. Phys. 1989, 90, 1007–1023. 10.1063/1.456153.

[ref33] Frisch M. J.; Trucks G. W.; Schlegel H. B.; Scuseria G. E.; Robb M. A.; Cheeseman J. R.; Scalmani G.; Barone V.; Petersson G. A.; Nakatsuji H.; Li X.; Caricato M.; Marenich A. V; Bloino J.; Janesko B. G.; Gomperts R.; Mennucci B.; Hratchian H. P.; Ortiz J. V; Izmaylov A. F.; Sonnenberg J. L.; Williams-Young D.; Ding F.; Lipparini F.; Egidi F.; Goings J.; Peng B.; Petrone A.; Henderson T.; Ranasinghe D.; Zakrzewski V. G.; Gao J.; Rega N.; Zheng G.; Liang W.; Hada M.; Ehara M.; Toyota K.; Fukuda R.; Hasegawa J.; Ishida M.; Nakajima T.; Honda Y.; Kitao O.; Nakai H.; Vreven T.; Throssell K.; Montgomery J. A. Jr.; Peralta J. E.; Ogliaro F.; Bearpark M. J.; Heyd J. J.; Brothers E. N.; Kudin K. N.; Staroverov V. N.; Keith T. A.; Kobayashi R.; Normand J.; Raghavachari K.; Rendell A. P.; Burant J. C.; Iyengar S. S.; Tomasi J.; Cossi M.; Millam J. M.; Klene M.; Adamo C.; Cammi R.; Ochterski J. W.; Martin R. L.; Morokuma K.; Farkas O.; Foresman J. B.; Fox D. J. Gaussian16; Revision B.01., Gaussian, Inc.: Wallingford CT, 2016.

[ref34] Zheng J.; Meana-Pañeda R.; Truhlar D. G. MSTor Version 2013: A New Version of the Computer Code for the Multi-Structural Torsional Anharmonicity, Now with a Coupled Torsional Potential. Comput. Phys. Commun. 2013, 184, 2032–2033. 10.1016/j.cpc.2013.03.011.

[ref35] Peng C.; Bernhard Schlegel H. Combining Synchronous Transit and Quasi-Newton Methods to Find Transition States. Isr. J. Chem. 1993, 33, 449–454. 10.1002/ijch.199300051.

[ref36] Zheng J.; Bao J. L.; Zhang S.; Corchado J. C.; Meana-Paneda R.; Chuang Y.-Y.; Coitino E. L.; Ellingson B. A.; Truhlar D. G. Gaussrate17; University of Minnesota: Minneapolis, MN, 2017.

[ref37] Zheng J.; Bao J. L.; Meana-Paneda R.; Zhang S.; Lynch B. J.; Corchado J. C.; Chuang Y.-Y.; Fast P. L.; Hu W.-P.; Liu Y.-P.; Lynch G. C.; Nguyen K. A.; Jackels C. F.; Fernandez-Ramos A.; Ellingson B. A.; Melissas V. S.; Villa J.; Rossi I.; Coitino E. L.; Pu J.; Albu T. V. Polyrate-Version 2016-2A; University of Minnesota: Minneapolis, MN, 2016.

[ref38] Page M.; McIver J. W. Jr. On Evaluating the Reaction Path Hamiltonian. J. Chem. Phys. 1988, 88, 922–935. 10.1063/1.454172.

[ref39] NIST. NIST Computational Chemistry Comparison and Benchmark Database; Johnson R. D. III, Ed.; NIST, Standard Reference Database Number 101, 2019. 10.18434/T47C7Z.

[ref40] Lu D.; Truong T. N.; Melissas V. S.; Lynch G. C.; Liu Y.-P.; Garrett B. C.; Steckler R.; Isaacson A. D.; Rai S. N.; Hancock G. C.; Lauderdale J. G.; Joseph T.; Truhlar D. G. POLYRATE 4: A New Version of a Computer Program for the Calculation of Chemical Reaction Rates for Polyatomics. Comput. Phys. Commun. 1992, 71, 235–262. 10.1016/0010-4655(92)90012-N.

[ref41] Fernandez-Ramos A.; Ellingson B. A.; Garrett B. C.; Truhlar D. G. Variational Transition State Theory with Multidimensional Tunneling. In Reviews in Computational Chemistry; Reviews in Computational Chemistry; Wiley Online Library, 2007; pp. 125–232. 10.1002/9780470116449.ch3.

[ref42] Fernández-Ramos A.; Miller J. A.; Klippenstein S. J.; Truhlar D. G. Modeling the Kinetics of Bimolecular Reactions. Chem. Rev. 2006, 106, 4518–4584. 10.1021/cr050205w. 17091928

[ref43] Zheng J.; Yu T.; Papajak E.; Alecu I. M.; Mielke S. L.; Truhlar D. G. Practical Methods for Including Torsional Anharmonicity in Thermochemical Calculations on Complex Molecules: The Internal-Coordinate Multi-Structural Approximation. Phys. Chem. Chem. Phys. 2011, 13, 10885–10907. 10.1039/C0CP02644A. 21562655

[ref44] Zheng J.; Truhlar D. G. Quantum Thermochemistry: Multistructural Method with Torsional Anharmonicity Based on a Coupled Torsional Potential. J. Chem. Theory Comput. 2013, 9, 1356–1367. 10.1021/ct3010722. 26587598

[ref45] Georgievskii Y.; Klippenstein S. J. Variable Reaction Coordinate Transition State Theory: Analytic Results and Application to the C2H3+H→C2H4 Reaction. J. Chem. Phys. 2003, 118, 5442–5455. 10.1063/1.1539035.

[ref46] Georgievskii Y.; Klippenstein S. J. Transition State Theory for Multichannel Addition Reactions: Multifaceted Dividing Surfaces. J. Phys. Chem. A 2003, 107, 9776–9781. 10.1021/jp034564b.

[ref47] Grajales-González E.; Monge-Palacios M.; Sarathy S. M. Collision Efficiency Parameter Influence on Pressure-Dependent Rate Constant Calculations Using the SS-QRRK Theory. J. Phys. Chem. A 2020, 124, 6277–6286. 10.1021/acs.jpca.0c02943. 32663402 PMC7458424

[ref48] Grajales-González E.; Monge-Palacios M.; Sarathy S. M. A Theoretical Study of the Ḣ- and HOȮ-Assisted Propen-2-Ol Tautomerizations: Reactive Systems to Evaluate Collision Efficiency Definitions on Chemically Activated Reactions Using SS-QRRK Theory. Combust. Flame 2021, 225, 485–498. 10.1016/j.combustflame.2020.11.015.

[ref49] Bao J. L.; Zheng J.; Truhlar D. G. Kinetics of Hydrogen Radical Reactions with Toluene Including Chemical Activation Theory Employing System-Specific Quantum RRK Theory Calibrated by Variational Transition State Theory. J. Am. Chem. Soc. 2016, 138, 2690–2704. 10.1021/jacs.5b11938. 26841076

[ref50] Bao J. L.; Zhang X.; Truhlar D. G. Predicting Pressure-Dependent Unimolecular Rate Constants Using Variational Transition State Theory with Multidimensional Tunneling Combined with System-Specific Quantum RRK Theory: A Definitive Test for Fluoroform Dissociation. Phys. Chem. Chem. Phys. 2016, 18, 16659–16670. 10.1039/C6CP02765B. 27273734

[ref51] Bao J. L.; Zhang X.; Truhlar D. G. Barrierless Association of CF2 and Dissociation of C2F4 by Variational Transition-State Theory and System-Specific Quantum Rice-Ramsperger-Kassel Theory. Proc. Natl. Acad. Sci. U. S. A. 2016, 113, 13606–13611. 10.1073/pnas.1616208113. 27834727 PMC5137698

[ref52] Rustemeier K.; Piadé J.-J. Chapter 12 - Determination of Nicotine in Mainstream and Sidestream Cigarette Smoke. In Analytical Determination of Nicotine and Related Compounds and their Metabolites; Gorrod J. W., Jacob P. of N. and R. C. and their M., Eds.; Elsevier Science: Amsterdam, 1999; pp. 489–529. 10.1016/B978-044450095-3/50013-9.

[ref53] Yoshida T.; Farone W. A.; Xantheas S. S. Isomers and Conformational Barriers of Gas-Phase Nicotine, Nornicotine, and Their Protonated Forms. J. Phys. Chem. B 2014, 118, 8273–8285. 10.1021/jp501646p. 24654683

[ref54] Robertson P. A.; Villani L.; Robertson E. G. Conformer Specific Ultraviolet and Infrared Detection of Nicotine in the Vapor Phase. J. Phys. Chem. A 2019, 123, 10152–10157. 10.1021/acs.jpca.9b09113. 31644291

[ref55] Egidi F.; Segado M.; Koch H.; Cappelli C.; Barone V. A Benchmark Study of Electronic Excitation Energies, Transition Moments, and Excited-State Energy Gradients on the Nicotine Molecule. J. Chem. Phys. 2014, 141, 224114 10.1063/1.4903307. 25494739

[ref56] Elmore D. E.; Dougherty D. A. A Computational Study of Nicotine Conformations in the Gas Phase and in Water. J. Org. Chem. 2000, 65, 742–747. 10.1021/jo991383q. 10814006

[ref57] Takeshima T.; Fukumoto R.; Egawa T.; Konaka S. Molecular Structure of Nicotine As Studied by Gas Electron Diffraction Combined with Theoretical Calculations. J. Phys. Chem. A 2002, 106, 8734–8740. 10.1021/jp020328+.

[ref58] Ortega P. G. R.; Montejo M.; González J. J. L. Vibrational Circular Dichroism and Theoretical Study of the Conformational Equilibrium in (−)-S-Nicotine. ChemPhysChem 2015, 16, 342–352. 10.1002/cphc.201402652. 25421493

[ref59] Kato K.; Osuka A. Platforms for Stable Carbon-Centered Radicals. Angew. Chem., Int. Ed. 2019, 58, 8978–8986. 10.1002/anie.201900307. 30746863

[ref60] Bartlett R. J. Coupled-Cluster Approach to Molecular Structure and Spectra: A Step toward Predictive Quantum Chemistry. J. Phys. Chem. 1989, 93, 1697–1708. 10.1021/j100342a008.

[ref61] Monge-Palacios M.; Rissanen M. P.; Wang Z.; Sarathy S. M. Theoretical Kinetic Study of the Formic Acid Catalyzed Criegee Intermediate Isomerization: Multistructural Anharmonicity and Atmospheric Implications. Phys. Chem. Chem. Phys. 2018, 20, 10806–10814. 10.1039/C7CP08538A. 29411814

[ref62] Monge-Palacios M.; Grajales-González E.; Sarathy S. M. Ab Initio, Transition State Theory, and Kinetic Modeling Study of the HO_2_-Assisted Keto–Enol Tautomerism Propen-2-Ol + HO_2_ ⇔ Acetone + HO_2_ under Combustion, Atmospheric, and Interstellar Conditions. J. Phys. Chem. A 2018, 122, 9792–9805. 10.1021/acs.jpca.8b10369. 30500199

[ref63] Monge-Palacios M.; Sarathy S. M. Ab Initio and Transition State Theory Study of the OH + HO_2_ → H_2_O + O_2_(3Σg−)/O_2_(1Δg) Reactions: Yield and Role of O_2_(1Δg) in H_2_O_2_ Decomposition and in Combustion of H2. Phys. Chem. Chem. Phys. 2018, 20, 4478–4489. 10.1039/C7CP05850K. 29372728

[ref64] Truhlar D. G.; Isaacson A. D.; Garrett B. C. Theory of Chemical Reaction Dynamics. Baer M., Ed; Springer Science & business Media, 1985, 4, 65–137.

[ref65] Monge-Palacios M.; Corchado J. C.; Espinosa-Garcia J. Dynamics Study of the OH + NH3 Hydrogen Abstraction Reaction Using QCT Calculations Based on an Analytical Potential Energy Surface. J. Chem. Phys. 2013, 138, 214306 10.1063/1.4808109. 23758370

[ref66] Passananti M.; Temussi F.; Iesce M. R.; Previtera L.; Mailhot G.; Vione D.; Brigante M. Photoenhanced Transformation of Nicotine in Aquatic Environments: Involvement of Naturally Occurring Radical Sources. Water Res. 2014, 55, 106–114. 10.1016/j.watres.2014.02.016. 24602865

[ref67] Egerton A.; Gugan K.; Weinberg F. J. The Mechanism of Smouldering in Cigarettes. Combust. Flame 1963, 7, 63–78. 10.1016/0010-2180(63)90156-1.

