# Supplementary material for: Early Chemistry of Nicotine Degradation in Heat-Not-Burn Smoking Devices and Conventional Cigarettes: Implications for Users and Second- and Third-Hand Smokers
Source: J Phys Chem A. 2021 Apr 9;125(15):3177–88. doi: 10.1021/acs.jpca.1c01650 (PMC8154610; doi:10.1021/acs.jpca.1c01650)
Supplement: Supplementary file 1 — jp1c01650_si_001.pdf [file jp1c01650_si_001.pdf]

Supporting Information

Early Chemistry of Nicotine Degradation in Heat-not-burn Smoking Devices and Conventional Cigarettes: Implications for Users and Second- and Third-Hand Smokers

Javier E. Chavarrio[a‡], M. Monge-Palacios*[a‡], E. Grajales-González[a], S. Mani Sarathy[a].

[a]Clean Combustion Research Center, Physical Science and Engineering (PSE) Division, King Abdullah University of Science and Technology, Al Kindi Building 5, Thuwal 23955, Saudi Arabia

In this supplementary material we provide further details about our calculations as well as additional results.

S1. Methods I: Multi-structural torsional variational transition state theory calculations

The final rate constant is computed with the following equation

$$k_{MS-T(C)}^{CVT/SCT}(T,s) = \kappa^{SCT}\Gamma\frac{k_B T}{h}\frac{Q_{el}^{\ddagger}Q_{con-rovib}^{MS-T(C),\ddagger}}{\Phi_t Q_{el}^{R} Q_{con-rovib}^{MS-T(C),R}}\exp\left(-\frac{V^{\ddagger}}{k_B T}\right) \quad (S1)$$

The superscripts ‡ and R represent the transition state and reactants, respectively, $V^{\ddagger}$ is the classical barrier height (difference between the potential energy of the global minimum of the transition state and reactants). The parameter κ^{SCT} is the transmission coefficient calculated with the SCT approach, which accounts for quantum effects in the reaction coordinate, and the factor Γ is the recrossing transmission coefficient (ratio of the CVT rate constant to the TST one). k_B and h are the Boltzmann's and Plank's constants, respectively, and T is temperature. The relative translational and the electronic partition functions are displayed as Φ_t and Q_{el}^{R} (or $Q_{el}^{\ddagger}$),

respectively; the electronically excited-state $^2\Pi_{1/2}$ of the radical OH, which lies 0.4 kcal mol^{-1} over the $^2\Pi_{3/2}$ ground-state, was included in the calculation of its electronic partition function. In Eq (S1), the conformational rovibrational partition function is defined as

$$Q_{con-rovib}^{MS-T(C),X} = \sum_{j=1}^{J} Q_{rot,j} \exp\left(-\frac{U_j}{k_B T}\right) Q_j^{HO} \prod_{\eta=1}^{t} \bar{f}_{j,\eta} \tag{S2}$$

where X represents either reactants (R) or saddle point (‡). The summation domain goes from j=1 to J, where J is the total number of distinguishable structures for the species X and j=1 is the global minimum. The term U_j is the potential energy of the conformer j with respect to that of the global minimum ($U_1 = 0$), and $Q_{rot,j}$ is the rotational partition function for the corresponding conformer. Q_j^{HO} stands for the normal-mode harmonic oscillator partition function, which is calculated as

$$Q_j^{HO} = \prod_{i=1}^{F} \frac{\exp\left(-\frac{h\omega_{j,i}}{2k_B T}\right)}{1 - \exp\left(-\frac{h\omega_{j,i}}{k_B T}\right)} \tag{S3}$$

where $\omega_{j,i}$ is the vibrational frequency of the normal-mode i of conformer j, and F the total number of normal modes. Finally, the term $\bar{f}_{j,\eta}$ is a factor to account for the torsional anharmonicity of the coupled torsion η of the conformer j, and t is the number of coupled torsions. This factor is calculated as follows

$$\prod_{\eta=1}^{t} \bar{f}_{j,\eta} = \left(\frac{h}{k_B T}\right)^{\frac{t}{2}} \frac{\prod_{m=1}^{F} \omega_{j,m}}{\prod_{\bar{\bar{m}}=1}^{F-t} \bar{\bar{\omega}}_{j,\bar{\bar{m}}}} \frac{\sqrt{\det \mathbf{D}_j}}{\prod_{\tau=1}^{t} M_{j,\tau}} \prod_{\eta=1}^{t} \exp\left(-\frac{W_{j,\eta}^{C}}{2k_B T}\right) I_0\left(\frac{W_{j,\eta}^{C}}{2k_B T}\right) \tag{S4}$$

where $\mathbf{D}_j$ is the Kilpatrick and Pitzer torsional moment of inertia matrix, $\bar{\bar{\omega}}_{j,\bar{\bar{m}}}$ the torsion-projected normal-mode frequencies and $M_{j,\tau}$ the local periodicity parameter of the torsion τ for the

conformer j. $W_{j,\eta}^{C}$ represents the energy barrier for the coupled torsion η in the conformer j. Lastly, I_0 is a modified Bessel function.

Torsions were divided into two types, nearly separable (NS) and strongly coupled (SC); for the latter, the Voronoi tessellation method [1,2] was used to calculate $M_{j,\tau}$. We treated torsions in all the species under the "Nearly Separable:Strongly Coupled" (NS:SC) scheme, as presented in the section S4 of this document.

Multi-structural torsional anharmonicity in the investigated reactions was estimated with the multi-structural torsional anharmonicity parameter, $F^{MS\text{-}T}$, which is obtained with the corresponding parameters for the saddle point and reactant species, denoted as $F^{MS-T,\ddagger}$ and $F^{MS-T,R}$, respectively. These parameters are calculated as follows, where X denotes ‡ or R for the saddle point and reactant, respectively, and $Q_{rovib}^{SS-T(C),X}$ is the torsional rovibrational partition function of the global minimum of the reactant or saddle point species

$$F^{MS-T} = \frac{F^{MS-T,\ddagger}}{F^{MS-T,R}} \quad \text{(S5)}$$

$$F^{MS-T,X} = \frac{Q_{con-rovib}^{MS-T(C),X}}{Q_{rovib}^{SS-T(C),X}} \quad \text{(S6)}$$

With the aim of investigate the effect of torsional anharmonicity in the rate constants, the parameter in Eq (S6) was also calculated using the harmonic partition function $Q_{rovib}^{SS-HO,X}$, yielding $F^{MS,X}$,

$$F^{MS,X} = \frac{Q_{con-rovib}^{MS-T(C),X}}{Q_{rovib}^{SS-HO,X}} \quad \text{(S7)}$$

Finally, the calculated rate constants within the temperature range 200-3000 K were fitted to the modified Arrhenius equation

$$k = A \cdot T^n \cdot \exp\left(-\frac{E_a}{RT}\right) \quad \text{(S8)}$$

where A (cm^3 $molecule^{-1}$ s^{-1}), n (unitless) and E_a (kcal mol^{-1}) are fitting parameters.

S2. Methods II: Variable reaction coordinate calculations

In radical-radical association reactions, the location of the dividing surface that minimizes the reactive flux shows some difficulties since the motion of one fragment relative to the other can be of large amplitude, and the separation between the two reacting fragments used to define that surface is very sensitive to the energy (E) and angular momentum (J). These shortcomings are overcome by the VRC-TST theory by optimizing not only the value of the reaction coordinate s, but also its definition, which is given by the distance between two pivot points which are located on each of the two reactants. The number of transitional states N(E,J,s) is minimized as a function of E, J and s, obtaining an E and J resolved reactive flux, $N(E,J,s)^{E,J\text{-}\mu VT}$, within the VRC-E,J-μVT scheme. In the VRC-TST formalism the normal modes are sorted into conserved and translational modes; the former are the vibrational modes of the reacting fragments, whose character barely changes during the reaction, while the latter are the rotation of the reacting fragments as well as their relative motion, which become vibrations and overall rotations as the association product is formed.

In both association reactions, the reaction coordinate was defined by a single-faceted dividing surface by using two pivot points, one for each radical fragment, which are located as shown in Figure S1 (black dots) for both association reactions. The distance d between each pivot point and its corresponding atom was initially set at 0.25 Å, and the distance between each pair of pivot points, r_{12} = s, was changed within the interval 1.6 Å ≤ 6.5 Å so that the reactive flux was variationally minimized by considering both parameters; the location of the pivot points was also varied during the minimization of the flux from d = 0.25 Å up to d = 0.35 Å. Intervals of 0.1 Å

and 0.01 Å were used to scan the parameters s and d, respectively, determining for each combination $N(E,J,s)^{E,J\text{-}\mu VT}$, which was used to calculate the variationally minimized rate constant as

$$k_{ass}^{E,J-\mu VT}(T,s) = \frac{\hbar^2}{2\pi} g_e \frac{\sigma_1\sigma_2}{\sigma^{\neq} Q_1 Q_2} \left(\frac{2\pi}{\mu k_B T}\right)^{3/2} \int \exp\left(-\frac{E}{k_B T}\right) N(E,J,s)^{E,J-\mu VT} dEdJ \quad \text{(S9)}$$

where g_e is the ratio of the electronic partition function of the transition state to the product of the electronic partition functions of the reactants, Q_1 and Q_2 are the rotational partition functions of the reactants without symmetry numbers, $\sigma^{\neq}$, σ_1 and σ_2 are the symmetry numbers for the transition state, reactant 1 and reactant 2, respectively, and $\hbar$ is the reduced Planck's constant.

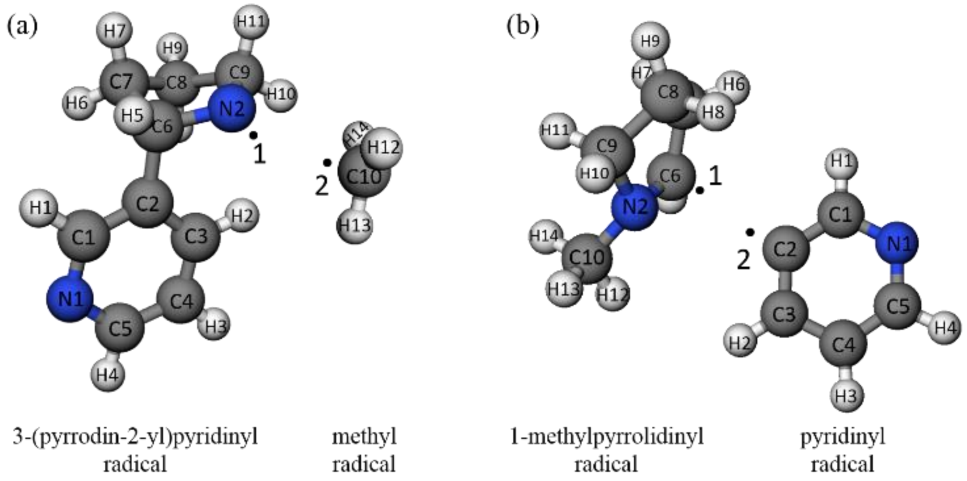

Figure S1. Location of the pivot points (black dots) that define the single-faceted dividing surface of reactions R_{ass}-CN (a) and R_{ass}-CC (b).

For each combination of d and s, 10200 configurations were explored by Monte Carlo integration to determine the flux $N(E,J,s)^{E,J\text{-}\mu VT}$, with a maximum angular momentum quantum number J equal to 450. The rate constants of the reaction R_{ass}-CN were multiplied by two to account for the two equivalent pathways that can form nicotine as the result of the approach of the planar methyl

radical along either of its two equivalent sides. These calculations were performed with the Gaussrate17 [3] and Polyrate 2016-2A [4] software.

For the electronic structure calculations the method MN15-L [5] and the basis set cc-pVTZ [6] were used. We used this local exchange-correlation functional for its favorable computational performance and accuracy ratio for energetic purposes; we tested the performance of the larger basis set aug-cc-pVTZ [6] in the prediction of the dissociation energy and dissociation curves of nicotine and similar results were obtained.

The rate constants for the reaction $nicotine + OH \rightarrow Reactant\ complex$ were also obtained with the VRC-TST formulation following a similar procedure to that previously explained. However, there were three important differences. First, the level of theory used was M06-2X/cc-pVTZ [6,7], that is, the same level that was used to characterize the PES of the hydrogen abstraction reactions. Second, the number of configurations for the Monte Carlo integration to determine the flux $N(E,J,s)^{E,J\text{-}\mu VT}$ was 8600 since these calculations were more computationally expensive than those for the radical-radical association reactions. And third, the pivot points located in the nicotine and OH fragments are now placed along the interaction axis that leads to the formation of the corresponding intermediate complex (see the reactant complex geometry of R-H11 in Figure S3); the distance d between the atom H9 of nicotine and the corresponding pivot point was varied from 1.95 Å to 1.80 Å with intervals of 0.0375 Å, while that for the atom O of the OH radical and its pivot point was varied from 0.4 Å to 0.1 Å, both with intervals of 0.075 Å. The distance between the pair of pivot points was changed within the interval 0.8-6.0 Å with intervals of 0.2 Å.

S3. Methods III: Pressure-dependent rate constant calculations. SS-QRRK/MSC approach

Applying the steady-state approximation to the energized nicotine molecule, that is, [nicotine]* (generated by collisions with the bath gas), the SS-QRRK/MSC method [8,9] yields the following pressure-dependent dissociation rate constant

$$k_{uni}(T,P) = \sum_{n=m}^{+\infty} \frac{k_1^{QRRK}(E = nhc\bar{v}; T)[M]}{1 + \frac{k_c(T)[M]}{k_2^{QRRK}(E = nhc\bar{v})}} \tag{S10}$$

where [M] is the concentration of the bath gas (obtained with the Redlich–Kwong equation of state), k_c is the collisional deactivation rate constant for the activated adduct [nicotine]*, $k_1^{QRRK}(E = nhc\bar{v}; T)$ is the rate constant for the thermal activation of nicotine, $k_2^{QRRK}(E = nhc\bar{v})$ is the rate constant for the dissociation of [nicotine]*, and E is the total rovibrational energy,

$$k_c(T) = Z\beta_c \tag{S11}$$

$$k_2^{QRRK}(E = nhc\bar{v}) = A^{QRRK} \frac{n!\,(n - m + s - 1)!}{(n - m)!\,(n + s - 1)!} \tag{S12}$$

$$k_1^{QRRK}(E = nhc\bar{v}; T) = \exp\left(\frac{-nhv}{k_b T}\right)\left[1 - \exp\left(\frac{-hv}{k_b T}\right)\right]^s \frac{(n + s - 1)!}{n!\,(s - 1)!} k_c(T) \tag{S13}$$

$$A^{QRRK} = k_{diss}(T)\exp\left(\frac{E_{diss}(T)}{RT}\right) \tag{S14}$$

$$m = E_{diss}(T)/hc\bar{v} \tag{S15}$$

A^{QRRK} is the frequency factor, Z and β_c are the Lennard-Jones collision rate constant and collision efficiency, respectively, n is the number of quanta excited at energy E, m is the number of quanta excited at energy E_o, s is the number of vibrational degrees of freedom of the adduct, and $E_{diss}(T)$ is the activation energy for the dissociation of the adduct into the reactants. Finally, the geometric mean frequency $\bar{v}$ was calculated as $\bar{v} = \left(\prod_{i=1}^{S} \nu_i\right)^{\frac{1}{S}}$, where ν_i is the frequency of the normal mode i, and s is the number of vibrational degrees of freedom.

Firstly, we obtained E_{diss} from the calculated $k_{ass}^{E,J-\mu VT}$ and the concentration equilibrium constant K_C^{diss}, and secondly, we fitted the HPL rate constants $k_{diss}(T)$ to the following equation

$$k = A\left(\frac{T}{300}\right)^n \exp\left[-\frac{E(T+T_o)}{R(T^2+T_o^2)}\right] \tag{S16}$$

The fitting parameters A, n, E, and T_o were used to calculate the threshold energy E_o, which is set equal to the HPL Arrhenius activation energy for the dissociation reaction which is calculated as

$$E_{diss}(T) = \frac{E(T^4 + 2T_oT^3 - T_o^2T^2)}{(T^2+T_o^2)^2} + nRT \tag{S17}$$

The values of E_{diss} are used to calculate A^{QRRK} and thus k_{stab}.

The Lennard-Jones collision rate constant was calculated with the Lennard-Jones parameters of nicotine; these parameters were estimated with the boiling point and density of nicotine, 520.15 K and 1.0097 g cm^{-3}, respectively, and the corresponding empirical correlations [10,11], obtaining values of σ = 6.307 Å and ε/k_B = 613.8 K. The bath gas was N_2, whose Lennard-Jones parameters are σ = 3.798 Å and ε/k_B = 71.4 K [12].

The collision efficiency was calculated as

$$\beta_c = \left(\frac{\langle\Delta E\rangle_{down}}{\langle\Delta E\rangle_{down} + F_E k_b T}\right)^2 \frac{1}{\Delta} \tag{S18}$$

where Δ is a correction factor implemented in our SS-QRRK/MSC formulation [13,14] which yields more accurate values for β_c, and F_E is the thermal fraction of unimolecular states above the threshold energy [15]. We used and tested two different definitions for Δ and thus for β_c in order to use the one that shows better performance; one was derived by Gilbert *et al.* [16] and the other one by Dean *et al.* [17,18]. The deactivation averaged energy transferred value $\langle\Delta E\rangle_{down}$ was calculated as follows

$$\langle \Delta E \rangle_{\text{down}} = \Theta \left(\frac{T}{300}\right)^n \qquad \text{(S19)}$$

where Θ = 200 cm^{-1} and n = 0.85 [19].

The pressure-dependent rate constants of the *nicotine + OH → Reactant complex → Products* reaction were obtained with the chemically activated mechanism using the SS-QRRK approach [8,9] with our recent implementation [13,14]. This methodology is convenient for this reaction because its PES presents only one reaction channel connecting the initial reactants *nicotine + OH* with the final products *nicotine radical + H_2O* through two connected intermediate complexes. The temperature and pressure ranges considered were 200-3000 K and 0.01-400 atm, respectively, using N_2 as the bath gas (Θ = 200 cm^{-1} and n = 0.85), with the same Lennard-Jones parameters as those used for the radical-radical association reactions.

The pressure-dependent rate constants for the stabilized reactant complex and products are given by

$$k_{\text{stab}} = k_{\text{ass,Complex}}^{\text{E,J}-\mu\text{VT}} \sum_{E_o}^{+\infty} \frac{k_c[M]f(E)}{k_c[M] + k_{\text{diss}}^{\text{QRRK}}(E) + k_2^{QRRK}(E)} \qquad \text{(S20)}$$

$$k_{Overall}^{SS-QRRK/MSC} = k_{\text{ass,Complex}}^{\text{E,J}-\mu\text{VT}} \sum_{E_o}^{+\infty} \frac{k_2^{QRRK}(E)f(E)}{k_c[M] + k_{\text{diss}}^{\text{QRRK}}(E) + k_2^{QRRK}(E)} \qquad \text{(S21)}$$

where f(E) is the fraction of the energized complex, which is given by

$$f(E) = \frac{k_{-1}^{QRRK}(E)K(E)}{\sum_{E_0}^{+\infty} k_{-1}^{QRRK}(E)K(E)} \qquad \text{(S22)}$$

K(E) is the equilibrium constant which has the form

$$K(E) = \exp\left(\frac{-nhv}{k_b T}\right)\left[1 - \exp\left(\frac{-hv}{k_b T}\right)\right]^s \frac{(n+s-1)!}{n!\,(s-1)!} \qquad \text{(S23)}$$

Considering the chemically activated PES, k_c can be obtained with Eq (S11) and the values of $k_2^{QRRK}(E)$ and $k_{diss}^{QRRK}(E)$ can be computed from Eq (S12) taking into account the Tolman activation energies (Eq (S17)) from the complex to the final products and from the complex back to the reactants (dissociation), respectively. The rest of the terms are equivalent to those described for the unimolecular process.

S4. Results I: Electronic structure calculations and NS:SC schemes

Figure S2 shows the potential energy barrier heights calculated by Borduas *et al.* [20,21] for the 14 possible abstraction sites at the M60-2X/6-311+G(d,p) level. The blue colored hydrogen atoms represent the reaction sites with positive energy barriers, which are expected to have markedly reduced reactivity compared to those with negative energy barriers and thus a marginal effect in nicotine oxidation. Most of those non-kinetically favored reactions correspond to hydrogen abstractions from the pyridine ring as they would lead to removal of aromaticity. We used these results to design our theoretical approach, as explained in the main text.

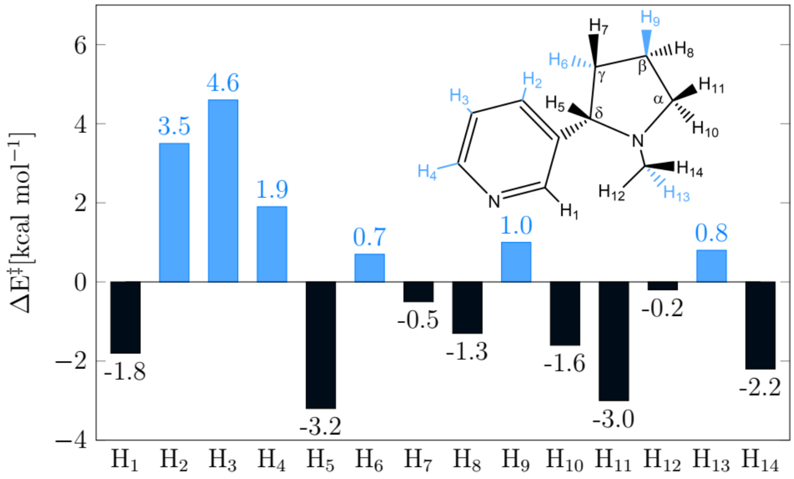

Figure S2. Classical potential energy barriers of the 14 possible hydrogen abstraction sites of nicotine by OH calculated by Borduas *et al.* [20] at the M60-2X/6-311+G(d,p) level. Abstraction sites with positive barriers are colored in blue.

The calculated MEPs for the investigated reactions are shown in Figure S3, including the optimized geometries of the corresponding stationary points. These MEPs converge to the reactant and product intermediate complexes in the entry and exit channels, respectively, whose optimized geometries are also shown.

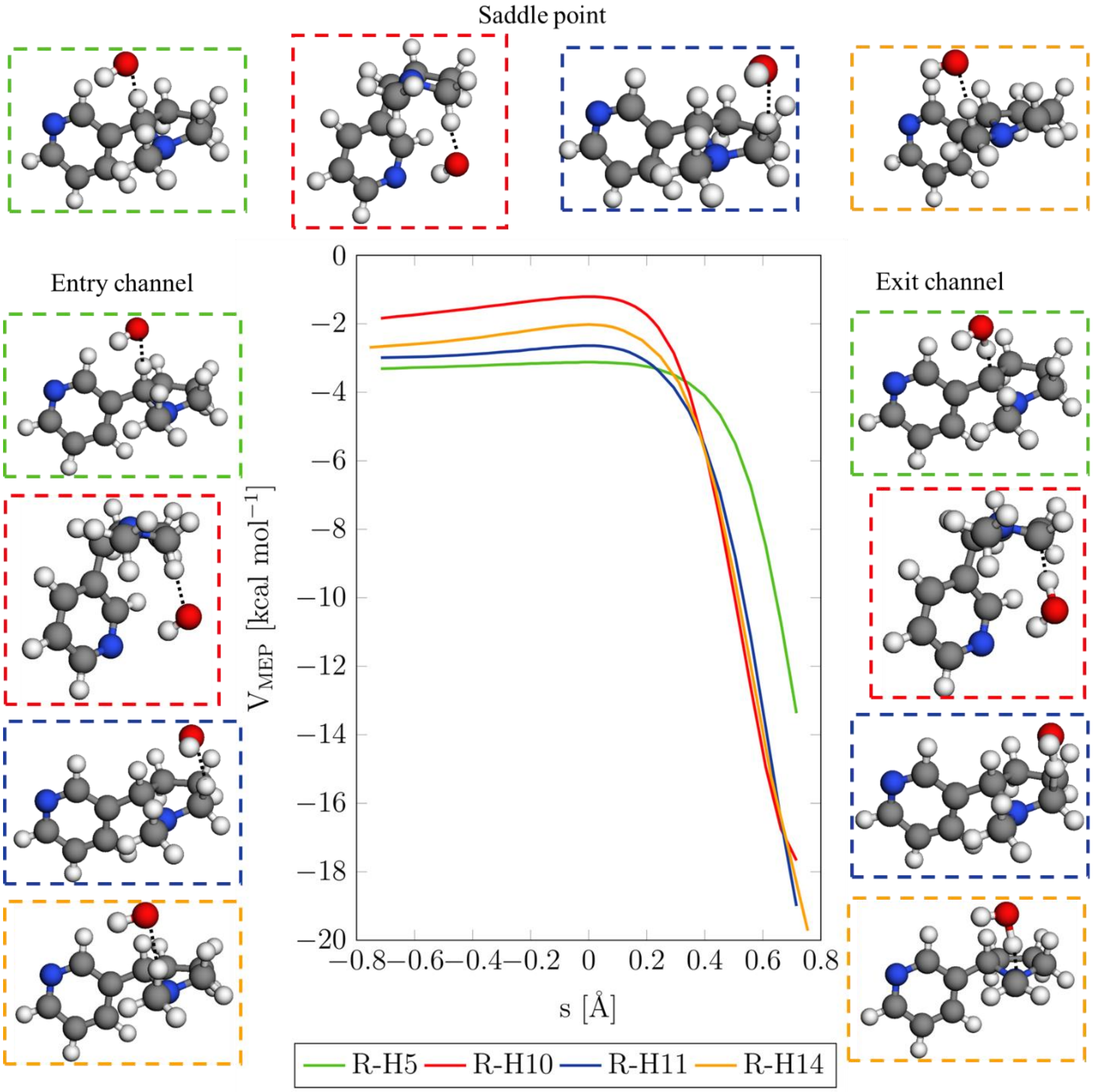

Figure S3. MEPs of reactions R-H5, R-H10, R-H11 and R-H14 at the M06-2X/aug-cc-pVQZ//M06-2X/cc-pVTZ level with the global minimum conformers of reactant and saddle point

species. Energies are defined with respect to that of the reactants. Both limits of the MEPs represent the formation of the reactant and product intermediate complexes, whose structures are also shown.

In Table S1 we describe the different NS:SC schemes used for each stationary point for the inclusion of multi-structural torsional anharmonicity, also indicating the involved torsions.

Table S1. Torsions and NS:SC schemes used in the treatment of multi-structural torsional anharmonicity.

Species	Torsion	Type	NS:SC
Nicotine	C1-C2-C6-H5	U	2:0
	C6-N2-C10-H14	U	
SP (R-H5) [a]	C1-C2-C6-H5	U	1:3
	C6-N2-C10-H14	C	
	C2-C6-H5-O1	C	
	C6-H5-O1-H15	C	
SP (R-H10) [a]	C1-C2-C6-H5	C	0:4
	C6-N2-C10-H14	C	
	C8-C9-H10-O1	C	
	C9-H10-O1-H15	C	
SP (R-H11) [a]	C1-C2-C6-H5	C	0:4
	C6-N2-C10-H14	C	
	C8-C9-H11-O1	C	
	C9-H11-O1-H15	C	
SP (R-H14) [a]	C1-C2-C6-H5	C	0:4

C6-N2-C10-H14	C
N2-C10-H14-O1	C
C10-H14-O1-H15	C

[a] SP: saddle point.

S5. Results II: Calculated rate constants

Table S2. Rate constants (cm^3 $molecule^{-1}$ s^{-1}) for the nicotine + OH hydrogen abstraction reactions calculated at the M06-2X/aug-cc-pVQZ//M06-2X/cc-pVTZ level of theory with multi-structural torsional anharmonicity and SCT corrections.

T(K)	k_{-R-H14}	k_{-R-H11}	k_{-R-H5}	$k_{-R-H110}$	$k_{R-overall}$
200	1.31E-11	1.85E-10	1.87E-10	5.30E-13	3.86E-10
250	6.40E-12	6.41E-11	4.60E-11	4.40E-13	1.17E-10
273	5.10E-12	4.55E-11	2.91E-11	4.18E-13	8.01E-11
278	4.88E-12	4.27E-11	2.66E-11	4.14E-13	7.46E-11
283	4.69E-12	4.00E-11	2.44E-11	4.11E-13	6.96E-11
288	4.50E-12	3.77E-11	2.25E-11	4.10E-13	6.51E-11
293	4.33E-12	3.55E-11	2.08E-11	4.07E-13	6.11E-11
298	4.18E-12	3.37E-11	1.93E-11	4.03E-13	5.76E-11
303	4.04E-12	3.19E-11	1.79E-11	4.00E-13	5.43E-11
308	3.91E-12	3.03E-11	1.67E-11	3.99E-13	5.13E-11
313	3.78E-12	2.88E-11	1.57E-11	3.98E-13	4.87E-11
350	3.08E-12	2.11E-11	1.02E-11	3.86E-13	3.47E-11
400	2.52E-12	1.54E-11	6.61E-12	3.85E-13	2.49E-11
450	2.18E-12	1.07E-11	4.85E-12	3.92E-13	1.81E-11
500	1.97E-12	9.10E-12	3.84E-12	4.02E-13	1.53E-11
550	1.83E-12	8.04E-12	3.23E-12	4.21E-13	1.35E-11
600	1.74E-12	7.34E-12	2.84E-12	4.41E-13	1.24E-11
650	1.68E-12	6.90E-12	2.57E-12	4.63E-13	1.16E-11
700	1.65E-12	6.59E-12	2.39E-12	4.90E-13	1.11E-11
750	1.64E-12	6.36E-12	2.27E-12	5.22E-13	1.08E-11
800	1.64E-12	6.24E-12	2.19E-12	5.55E-13	1.06E-11
850	1.66E-12	6.17E-12	2.13E-12	5.90E-13	1.06E-11
900	1.69E-12	6.15E-12	2.10E-12	6.30E-13	1.06E-11
950	1.72E-12	6.17E-12	2.09E-12	6.72E-13	1.07E-11
1000	1.75E-12	6.23E-12	2.09E-12	7.18E-13	1.08E-11
1100	1.81E-12	6.39E-12	2.12E-12	8.17E-13	1.11E-11

1200	1.89E-12	6.61E-12	2.19E-12	9.26E-13	1.16E-11
1300	1.99E-12	6.91E-12	2.28E-12	1.05E-12	1.22E-11
1400	2.11E-12	7.25E-12	2.38E-12	1.18E-12	1.29E-11
1500	2.24E-12	7.60E-12	2.52E-12	1.32E-12	1.37E-11
1600	2.39E-12	8.01E-12	2.66E-12	1.48E-12	1.45E-11
1700	2.54E-12	7.88E-12	2.81E-12	1.65E-12	1.49E-11
1800	2.69E-12	8.33E-12	2.99E-12	1.83E-12	1.58E-11
1900	2.87E-12	8.81E-12	3.17E-12	2.02E-12	1.69E-11
2000	3.05E-12	9.30E-12	3.35E-12	2.22E-12	1.79E-11
2200	3.43E-12	1.04E-11	3.76E-12	2.64E-12	2.02E-11
2400	3.86E-12	1.15E-11	4.19E-12	3.12E-12	2.26E-11
2600	4.29E-12	1.27E-11	4.68E-12	3.63E-12	2.53E-11
2800	4.77E-12	1.39E-11	4.56E-12	4.18E-12	2.74E-11
3000	5.26E-12	1.52E-11	5.05E-12	4.75E-12	3.03E-11

Table S3. Fitting parameters of the modified Arrhenius equation for the reactions R-H5, R-H10, R-H11, R-H14, and R-overall ($R^2 > 0.99$).

Reaction	A [cm^3 $molecule^{-1}$ s^{-1}]	n	E_a [kcal mol^{-1}]
R-H5	3.263×10^{-19}	1.992	-3.863
R-H10	1.419×10^{-19}	2.124	-1.613
R-H11	9.987×10^{-18}	1.707	-3.120
R-H14	7.157×10^{-18}	1.628	-2.356
R-overall	3.008×10^{-18}	1.941	-3.367

Table S4. Rate constants (cm^3 $molecule^{-1}$ s^{-1}) for the nicotine + OH hydrogen abstraction reactions calculated with the CUS (high-pressure limit) and SS-QRRK/MSC (high-pressure limit and 1 atm) approaches for reaction R-H11.

T(K)	$k_{Overall}^{SS-QRRK/MSC}$(1 atm)[a]	$k_{Overall}^{SS-QRRK/MSC}$(HPL)	k_{R-H11}^{CUS}
200	3.7463E-10	1.9996E-10	1.8377E-10
250	2.1049E-10	7.9183E-11	6.9813E-11
273	1.7004E-10	6.7157E-11	4.948E-11
278	1.6288E-10	6.4987E-11	4.6372E-11
283	1.562E-10	6.2943E-11	4.3337E-11
288	1.4995E-10	6.1016E-11	4.0739E-11

293	1.441E-10	5.9197E-11	3.8261E-11
298	1.3862E-10	5.7475E-11	3.6226E-11
303	1.3347E-10	5.5846E-11	3.4186E-11
308	1.2863E-10	5.4304E-11	3.2363E-11
313	1.2407E-10	5.284E-11	3.0656E-11
350	9.7475E-11	4.4025E-11	2.1884E-11
400	7.4598E-11	3.6001E-11	1.5421E-11
450	6.0135E-11	3.0612E-11	1.0424E-11
500	5.0438E-11	2.6822E-11	8.6528E-12
550	4.3638E-11	2.4071E-11	7.486E-12
600	3.8696E-11	2.2023E-11	6.7086E-12
650	3.4998E-11	2.0803E-11	6.1988E-12
700	3.2161E-11	1.9549E-11	5.8295E-12
750	2.9944E-11	1.8568E-11	5.5487E-12
800	2.818E-11	1.779E-11	5.3706E-12
850	2.6755E-11	1.7166E-11	5.2432E-12
900	2.5589E-11	1.6663E-11	5.1625E-12
950	2.4623E-11	1.6253E-11	5.1176E-12
1000	2.3816E-11	1.5918E-11	5.1059E-12
1100	2.2552E-11	1.5414E-11	5.1207E-12
1200	2.1619E-11	1.5064E-11	5.1843E-12
1300	2.0909E-11	1.4817E-11	5.3015E-12
1400	2.0355E-11	1.4637E-11	5.4425E-12
1500	1.9913E-11	1.4505E-11	5.5876E-12
1600	1.9554E-11	1.4405E-11	5.7616E-12
1700	1.9256E-11	1.4331E-11	5.6578E-12
1800	1.9005E-11	1.4275E-11	5.8529E-12
1900	1.8789E-11	1.4234E-11	6.0551E-12
2000	1.8602E-11	1.4202E-11	6.2538E-12
2200	1.829E-11	1.4159E-11	6.6776E-12
2400	1.8031E-11	1.4129E-11	7.0639E-12
2600	1.7801E-11	1.4096E-11	7.4498E-12
3000	1.7386E-11	1.4001E-11	8.1403E-12

[a] At 0.01, 0.1 and 1.0 atm, similar values for $k_{Overall}^{SS-QRRK/MSC}$ were obtained

Table S5. Rate constants for the nicotine association and dissociation *via* C-C bond calculated at the MN15-L/cc-pVTZ level of theory. The concentration equilibrium dissociation constants are also tabulated.

T (K)	k_{ass} (cm^3 $molecule^{-1}$ s^{-1})	k_{diss} (s^{-1})	Kc_{diss} (cm^{-3} molecule)
200	3.24E-16	1.81E-89	5.58E-74
250	8.83E-16	1.81E-68	2.05E-53
298	1.81E-15	6.54E-55	3.60E-40
350	3.43E-15	2.16E-44	6.31E-30
400	5.68E-15	7.70E-37	1.36E-22
450	8.89E-15	6.00E-31	6.75E-17
500	1.36E-14	3.26E-26	2.40E-12
550	2.04E-14	2.56E-22	1.25E-08
600	3.03E-14	4.68E-19	1.55E-05
650	4.40E-14	2.77E-16	6.30E-03
700	6.26E-14	6.73E-14	1.08E+00
750	8.68E-14	7.94E-12	9.14E+01
800	1.18E-13	5.21E-10	4.41E+03
850	1.57E-13	2.10E-08	1.34E+05
900	2.04E-13	5.62E-07	2.75E+06
950	2.62E-13	1.07E-05	4.08E+07
1000	3.30E-13	1.52E-04	4.59E+08
1050	4.08E-13	1.66E-03	4.08E+09
1100	4.95E-13	1.46E-02	2.95E+10
1200	6.95E-13	6.42E-01	9.24E+11
1300	9.25E-13	1.55E+01	1.67E+13
1400	1.18E-12	2.33E+02	1.97E+14
1500	1.46E-12	2.40E+03	1.65E+15
1600	1.76E-12	1.83E+04	1.04E+16
1700	2.09E-12	1.10E+05	5.27E+16
1800	2.45E-12	5.40E+05	2.20E+17
1900	2.86E-12	2.24E+06	7.83E+17
2000	3.30E-12	8.05E+06	2.44E+18
2100	3.79E-12	2.56E+07	6.76E+18
2200	4.31E-12	7.33E+07	1.70E+19
2300	4.87E-12	1.91E+08	3.92E+19
2400	5.44E-12	4.56E+08	8.39E+19
2500	6.03E-12	1.01E+09	1.68E+20
2600	6.63E-12	2.11E+09	3.18E+20
2700	7.24E-12	4.14E+09	5.72E+20

2800	6.67E-12	6.55E+09	9.82E+20
2900	6.02E-12	9.74E+09	1.62E+21
3000	5.45E-12	1.40E+10	2.57E+21

Table S6. Rate constants for the nicotine association and dissociation *via* C-N bond calculated at the MN15-L/cc-pVTZ level of theory. The concentration equilibrium dissociation constants are also tabulated.

T (K)	k_{ass} (cm^3 molecule^{-1} s^{-1})	k_{diss} (s^{-1})	Kc_{diss} (cm^{-3} molecule)
200	2.39E-15	2.73E-69	1.14E-54
250	3.27E-15	1.61E-52	4.93E-38
298	4.13E-15	1.10E-41	2.65E-27
350	5.10E-15	2.77E-33	5.44E-19
400	6.08E-15	3.00E-27	4.94E-13
450	7.12E-15	1.51E-22	2.13E-08
500	8.27E-15	8.93E-19	1.08E-04
550	9.55E-15	1.10E-15	1.16E-01
600	1.10E-14	4.23E-13	3.83E+01
650	1.27E-14	6.56E-11	5.16E+03
700	1.46E-14	5.01E-09	3.42E+05
750	1.68E-14	2.16E-07	1.28E+07
800	1.93E-14	5.89E-06	3.04E+08
850	2.22E-14	1.09E-04	4.93E+09
900	2.53E-14	1.47E-03	5.81E+10
950	2.88E-14	1.51E-02	5.25E+11
1000	3.27E-14	1.24E-01	3.78E+12
1050	3.70E-14	8.30E-01	2.25E+13
1100	4.16E-14	4.69E+00	1.13E+14
1200	5.23E-14	9.76E+01	1.87E+15
1300	6.48E-14	1.28E+03	1.97E+16
1400	7.95E-14	1.17E+04	1.47E+17
1500	9.67E-14	7.97E+04	8.24E+17
1600	1.17E-13	4.30E+05	3.68E+18
1700	1.40E-13	1.91E+06	1.37E+19
1800	1.66E-13	7.22E+06	4.34E+19
1900	1.97E-13	2.38E+07	1.21E+20
2000	2.31E-13	6.99E+07	3.03E+20
2100	2.69E-13	1.85E+08	6.90E+20
2200	3.11E-13	4.51E+08	1.45E+21

2300	3.58E-13	1.01E+09	2.83E+21
2400	4.10E-13	2.13E+09	5.21E+21
2500	4.65E-13	4.23E+09	9.09E+21
2600	5.25E-13	7.95E+09	1.51E+22
2700	5.90E-13	1.43E+10	2.42E+22
2800	6.58E-13	2.45E+10	3.72E+22
2900	7.31E-13	4.04E+10	5.53E+22
3000	8.07E-13	6.45E+10	7.99E+22

Table S7. Fitting parameters of the modified Arrhenius equation for the reactions R_{diss}-CN, R_{ass}-CN, R_{diss}-CC and R_{ass}-CC. Units of A for the association and dissociation reactions are cm^3 $molecule^{-1}$ s^{-1} and s^{-1}, respectively.

Reaction	A	n	Ea [kcal mol^{-1}]	R^2
Rass-CC	4.056×10^{-22}	3.023	1.113	0.891
Rass-CN	3.935×10^{-24}	3.217	-1.368	0.999
Rdiss-CC	1.360×10^{15}	0.693	96.764	0.967
Rdiss-CN	3.935×10^{11}	1.386	76.315	1.000

S6. Results III: Optimized molecular structures

In this section we provide the Cartesian coordinates matrix of the optimized structures for the conformers of the nicotine and the saddle point species of the studied reactions. Those for water, hydroxyl radical and products are not presented. All the geometries were optimized at the M06-2X/cc-pVTZ level, and the nature of the stationary points was corroborated by their vibrational frequencies. The displayed relative potential energies (with respect to the energy of the global minimum) were computed at the M06-2X/aug-cc-pVQZ//M06-2X/cc-pVTZ single point level.

Nicotine conformers

Conformer 1. ΔE=5.31 kcal mol^{-1}

6	1.43526656	-1.01771948	-0.55310891
1	0.89814854	-1.88349455	-0.92599284
6	0.72612430	0.15724912	-0.29409393
6	1.44785117	1.23866950	0.18632304
1	0.95463193	2.17818022	0.39865970
6	2.81476499	1.10689730	0.38828651
1	3.40558286	1.93159929	0.76116578
6	3.41264130	-0.10839486	0.10284335
1	4.47721074	-0.24723865	0.25021788
7	2.73993950	-1.16137960	-0.36104162
6	-0.74884495	0.21664948	-0.58513259
1	-0.86187464	0.24224548	-1.67518495
6	-1.53031112	1.41949049	-0.01778267
1	-1.18408171	1.65783195	0.98832277
1	-1.40027372	2.31184214	-0.62763141
6	-2.98801887	0.91450406	0.00752090
1	-3.41707878	1.01514252	1.00293133
1	-3.62858979	1.46701613	-0.67677879
6	-2.87494537	-0.57542936	-0.40846875
1	-3.56693911	-1.22689728	0.12133868
1	-3.06741039	-0.67830143	-1.47768504
7	-1.49209005	-0.97792031	-0.15009575
6	-1.29613119	-1.28911169	1.26277343
1	-0.25900440	-1.56178811	1.44661497
1	-1.55663554	-0.46289552	1.93908796
1	-1.91901315	-2.14497014	1.51793336

Conformer 2. ΔE=4.99 kcal mol^{-1}

6	1.45615959	1.21197059	0.17807940
1	0.95981092	2.15272705	0.38670121
6	0.72770054	0.12936747	-0.29981044
6	1.42937273	-1.04512698	-0.55866082
1	0.89335181	-1.91029136	-0.92888314
6	2.79036879	-1.08617876	-0.32430991
1	3.36418314	-1.98326809	-0.50971658
6	3.41545888	0.05811663	0.15746942
1	4.48181225	0.06132650	0.35179858
7	2.76950898	1.19068510	0.40409047
6	-0.74705656	0.20925708	-0.58442609
1	-0.86241774	0.24466685	-1.67405268
6	-1.51934533	1.41261717	-0.00642041
1	-1.17444046	1.63841459	1.00267287

1	-1.37999330	2.30998929	-0.60641678
6	-2.98128461	0.91874520	0.00778075
1	-3.41525571	1.01693506	1.00128766
1	-3.61354158	1.48073627	-0.67654483
6	-2.87887201	-0.56927761	-0.41758299
1	-3.57655638	-1.21885600	0.10717208
1	-3.07003237	-0.66451651	-1.48770369
7	-1.49892902	-0.98405873	-0.15896250
6	-1.31149007	-1.30068922	1.25439607
1	-0.27449410	-1.56928016	1.44477471
1	-1.57905018	-0.47826087	1.93199021
1	-1.93350767	-2.15951663	1.50193477

Conformer 3. ΔE=0.00 kcal mol^{-1}

6	-1.65149322	-0.34427704	-1.19539207
1	-1.31973043	-0.55775107	-2.20714851
6	-0.71031236	-0.14647250	-0.19297645
6	-1.18369328	0.14262062	1.08189364
1	-0.47653482	0.32228455	1.88211705
6	-2.54887433	0.21074404	1.29186873
1	-2.95518861	0.43693789	2.26771664
6	-3.39963087	-0.01652340	0.21669011
1	-4.47456039	0.02745395	0.34846940
7	-2.96833158	-0.28868584	-1.00986596
6	0.76228248	-0.26864669	-0.46946129
1	0.91653835	-0.21857061	-1.56259956
6	1.39152989	-1.56622910	0.06542213
1	0.87568502	-1.86482791	0.97690278
1	1.29208029	-2.37865253	-0.65138936
6	2.85890880	-1.18828526	0.36428337
1	3.09675603	-1.37882849	1.40850004
1	3.56551613	-1.75007278	-0.24248139
6	2.92739952	0.31124527	0.05743054
1	3.58175528	0.86630643	0.72894540
1	3.28648022	0.47318610	-0.97285751
7	1.55094798	0.76560953	0.19073969
6	1.33620519	2.09101080	-0.34944093
1	0.29482871	2.38387856	-0.22003040
1	1.96315499	2.80864942	0.17867051
1	1.58097356	2.14042021	-1.42283789

Conformer 4. ΔE=0.55 kcal mol^{-1}

6	1.19017919	0.18711509	-1.04498502
1	0.47954207	0.40378262	-1.83499110

6	0.71359116	-0.15503284	0.22013698
6	1.65240853	-0.40097207	1.20942789
1	1.32857848	-0.65802726	2.21117037
6	3.00169700	-0.31051843	0.90304832
1	3.75997330	-0.49608521	1.65053703
6	3.36272891	0.03303985	-0.38996509
1	4.40754377	0.11559723	-0.66568003
7	2.47829083	0.28323807	-1.35402266
6	-0.76314006	-0.28122991	0.47662652
1	-0.92772474	-0.25667042	1.56986331
6	-1.38700413	-1.56707017	-0.09296076
1	-0.86347683	-1.84437198	-1.00661578
1	-1.29357509	-2.39558626	0.60621253
6	-2.85210611	-1.18249763	-0.39500762
1	-3.07944234	-1.34537509	-1.44614090
1	-3.56400858	-1.76039306	0.19011797
6	-2.92424938	0.30849277	-0.05032637
1	-3.57524561	0.87996798	-0.71113574
1	-3.28908145	0.44376270	0.98210557
7	-1.54785682	0.76667974	-0.16436807
6	-1.33637454	2.08048126	0.40334787
1	-0.29536631	2.37791654	0.28168533
1	-1.96288226	2.80787784	-0.11162276
1	-1.58425577	2.10733225	1.47717297

Conformer 5. $\Delta E = 2.63$ kcal mol^{-1}

6	1.56229535	0.89054794	0.76644897
1	1.32788365	1.75845781	1.37662333
6	0.55177885	-0.01892290	0.46145068
6	0.91476229	-1.11910082	-0.30823816
1	0.18456047	-1.87661482	-0.56441784
6	2.22632335	-1.24908956	-0.73531103
1	2.53797038	-2.09387728	-1.33326833
6	3.14324264	-0.27354279	-0.37294832
1	4.17759503	-0.34589769	-0.68808222
7	2.82609566	0.78232031	0.37092853
6	-0.86796634	0.25051401	0.92123249
1	-0.81524306	0.89330710	1.80263129
6	-1.68294010	-1.02050035	1.19443176
1	-1.05335489	-1.84503907	1.52293480
1	-2.40056062	-0.81581177	1.98532942
6	-2.42455974	-1.31431249	-0.13242537
1	-2.09301305	-2.24274266	-0.59525978
1	-3.49410467	-1.40628216	0.04169351
6	-2.12000775	-0.10295836	-1.02411865
1	-1.32688840	-0.33835735	-1.75176862

1	-2.98579446	0.24314826	-1.58923422
7	-1.69809013	0.92365451	-0.08548490
6	-1.13240489	2.10037723	-0.70479559
1	-0.83052822	2.81368079	0.06294629
1	-0.25745696	1.88121790	-1.33412490
1	-1.89024588	2.57491567	-1.32846885

Conformer 6. ΔE=2.78 kcal mol^{-1}

6	0.92861378	-1.09774105	-0.29131195
1	0.19537632	-1.85725854	-0.54126618
6	0.55802813	0.00635837	0.47583171
6	1.56066965	0.92084763	0.77505956
1	1.32945036	1.79265651	1.37666201
6	2.84711658	0.71031417	0.30890843
1	3.64578181	1.40360291	0.53199175
6	3.09384069	-0.42122997	-0.45446508
1	4.08720379	-0.62045618	-0.83923457
7	2.15688136	-1.31637989	-0.75208121
6	-0.86999961	0.25336695	0.92726470
1	-0.82884667	0.88985650	1.81421271
6	-1.67516529	-1.02710466	1.18803216
1	-1.04041631	-1.84925899	1.51193012
1	-2.39599469	-0.83297434	1.97877719
6	-2.41176613	-1.31690148	-0.14222495
1	-2.07391963	-2.24080433	-0.60880995
1	-3.48153814	-1.41303363	0.02843885
6	-2.10850480	-0.09988258	-1.02603692
1	-1.30773651	-0.32744805	-1.74714618
1	-2.97200042	0.24370064	-1.59601537
7	-1.70089116	0.92553108	-0.07853151
6	-1.14410820	2.11133842	-0.68719277
1	-0.85774896	2.82468128	0.08695000
1	-0.26160645	1.90620204	-1.31071997
1	-1.90228478	2.58028112	-1.31467075

Conformer 7. ΔE=5.54 kcal mol^{-1}

6	1.47699623	-1.26017047	-0.24618706
1	1.03373501	-2.23781306	-0.40860689
6	0.67921420	-0.12412505	-0.38673262
6	1.28291792	1.10001377	-0.14820979
1	0.72050048	2.02132744	-0.23105796
6	2.62504655	1.13901143	0.20608223
1	3.12309884	2.07822032	0.40114473
6	3.31982915	-0.05375403	0.30385693
1	4.36987513	-0.06002315	0.57146813

7	2.76111713	-1.24354528	0.08165462
6	-0.78250805	-0.30905787	-0.73871290
1	-0.84519139	-1.05143207	-1.53546955
6	-1.52051494	0.99302720	-1.11266556
1	-0.88371828	1.70441798	-1.63360108
1	-2.34349078	0.75705813	-1.78487527
6	-2.05781292	1.51842316	0.23597677
1	-1.57737415	2.44769903	0.53897672
1	-3.12686423	1.71518894	0.17858944
6	-1.74227418	0.37749363	1.23758095
1	-0.83766812	0.61288467	1.79926779
1	-2.54158253	0.19657539	1.95349661
7	-1.49564501	-0.82697102	0.44088336
6	-2.73963646	-1.48915352	0.07265330
1	-2.52086186	-2.33689608	-0.57568191
1	-3.22104646	-1.86939856	0.97250495
1	-3.45526150	-0.83444446	-0.44577516

Conformer 8. ΔE=4.46 kcal mol^{-1}

6	1.63115612	1.09605883	-0.49968467
1	1.30572253	2.06236052	-0.87316668
6	0.70412294	0.06853913	-0.33494450
6	1.18821594	-1.15002916	0.11921071
1	0.50279119	-1.97858804	0.23594096
6	2.53872218	-1.27858008	0.40327639
1	2.94543350	-2.21281993	0.76450435
6	3.36770357	-0.18502351	0.20970603
1	4.42861481	-0.25157310	0.42046963
7	2.92965300	0.98783256	-0.24199636
6	-0.75408327	0.28138083	-0.69964745
1	-0.81183148	0.36346291	-1.78661211
6	-1.37350983	1.53598868	-0.02014947
1	-0.61526787	2.26288321	0.25938638
1	-2.05931817	2.03108625	-0.70582616
6	-2.12956256	0.97849586	1.20464733
1	-1.76267389	1.39402156	2.14056657
1	-3.19161230	1.21012090	1.14165261
6	-1.88824093	-0.54367615	1.12982471
1	-1.02483149	-0.81232789	1.74046365
1	-2.73524838	-1.13770650	1.46818001
7	-1.58336305	-0.84784282	-0.27075458
6	-2.79519194	-0.95530564	-1.07123601
1	-3.41887619	-0.04981927	-1.04873438
1	-2.52895898	-1.15959003	-2.10737970
1	-3.39396623	-1.78853148	-0.70620703

Saddle point R-H5 conformers

Conformer 1. ΔE=6.13 kcal mol^{-1}

6	-1.48686853	0.98132445	0.39808735
1	-0.90988817	1.83813939	0.72981094
6	-0.82142317	-0.10947689	-0.16723333
6	-1.60034905	-1.17769673	-0.58850052
1	-1.14293536	-2.04394172	-1.04792450
6	-2.97555808	-1.12156296	-0.41896449
1	-3.61003433	-1.93700026	-0.73587253
6	-3.52599967	0.00566250	0.16760857
1	-4.59631912	0.08201332	0.31788000
7	-2.79895340	1.04658528	0.57301954
6	0.66063326	-0.06636390	-0.35685357
1	0.82742200	0.65089082	-1.24014821
6	1.38772233	-1.37265969	-0.72295961
1	0.95018982	-2.20589821	-0.17006700
1	1.29847015	-1.60109765	-1.78316750
6	2.84257057	-1.11997098	-0.27962605
1	3.15550643	-1.86045242	0.45454375
1	3.54237549	-1.16981712	-1.11039184
6	2.80548160	0.29792146	0.33552071
1	3.47129571	0.42121084	1.18687250
1	3.07315969	1.03302931	-0.42313110
7	1.41394542	0.54026914	0.73924559
6	1.12745318	-0.06849056	2.03781667
1	0.09237447	0.11117280	2.31838872
1	1.30376032	-1.15260118	2.05416530
1	1.76743706	0.39436603	2.78668813
8	1.10224265	2.05639033	-1.76274905
1	1.17232590	2.38876218	-0.85088461

Conformer 2. ΔE=5.87 kcal mol^{-1}

6	-1.60111266	-1.15972576	-0.56528488
1	-1.13743276	-2.02990158	-1.01489479
6	-0.82100083	-0.08093998	-0.16211097
6	-1.48387069	1.01496679	0.38614992
1	-0.91361381	1.87966161	0.70126185
6	-2.85710240	0.97531679	0.52330289
1	-3.40194729	1.80664062	0.94763180
6	-3.53407126	-0.16062597	0.09393698
1	-4.61219503	-0.22445795	0.18433496
7	-2.92624939	-1.21059375	-0.44407391
6	0.66035886	-0.05040499	-0.35858192
1	0.82825283	0.69168385	-1.22346274

6	1.37906871	-1.35109072	-0.75603484
1	0.94427780	-2.19297454	-0.21495296
1	1.27864973	-1.56012167	-1.81904462
6	2.83851241	-1.11310477	-0.31969193
1	3.15839434	-1.87648361	0.38745792
1	3.52956126	-1.13770676	-1.15888976
6	2.81074641	0.28543094	0.33829293
1	3.47728127	0.37833092	1.19298375
1	3.08481236	1.04111878	-0.39759692
7	1.42049770	0.52625589	0.75035885
6	1.13312676	-0.11438198	2.03349158
1	0.09554098	0.05250699	2.31296152
1	1.31480773	-1.19747475	2.02443158
1	1.76803263	0.33361593	2.79567241
8	1.09433477	2.12036155	-1.67429065
1	1.22322174	2.39039250	-0.74838984

Conformer 3. ΔE=0.00 kcal mol^{-1}

6	-1.65516460	0.99929359	-0.49666653
1	-1.22589180	1.93637129	-0.83963724
6	-0.81314486	-0.05097527	-0.14781184
6	-1.40306484	-1.23058020	0.29137742
1	-0.77393441	-2.06351254	0.57969762
6	-2.78273539	-1.30491539	0.35857680
1	-3.27914491	-2.20294298	0.69858376
6	-3.52823402	-0.19535165	-0.02025383
1	-4.61093898	-0.22177657	0.02040348
7	-2.98350691	0.94122678	-0.44134636
6	0.67630832	0.07787786	-0.28490291
1	0.93034647	1.17808037	-0.26120820
6	1.25597839	-0.48946385	-1.59162166
1	0.64472820	-1.32630608	-1.92729419
1	1.24610470	0.26422183	-2.37603718
6	2.67852441	-0.95348014	-1.20942820
1	2.77587134	-2.02832873	-1.34645200
1	3.44900779	-0.47035720	-1.80520354
6	2.80399124	-0.59094345	0.27174061
1	3.40901272	-1.29187988	0.84560289
1	3.23773706	0.41483424	0.38698954
7	1.42650262	-0.59355129	0.74872833
6	1.26842135	-0.02257573	2.06833693
1	0.22393616	-0.06913090	2.37477907
1	1.86346504	-0.58529816	2.78629396
1	1.59833211	1.02835415	2.08819241
8	1.23184054	2.83388424	0.05532981
1	0.55039421	2.89955412	0.74489675

Conformer 4. ΔE=0.16 kcal mol^{-1}

6	-1.38306351	-1.20913448	0.31830270
1	-0.74265845	-2.02586087	0.63316065
6	-0.80196346	-0.03426340	-0.15628979
6	-1.65148340	0.99400352	-0.53896031
1	-1.23596759	1.92673862	-0.90273019
6	-3.02144917	0.80768925	-0.44180482
1	-3.71408658	1.58510733	-0.73177006
6	-3.49206152	-0.40269647	0.04429191
1	-4.55691689	-0.58120850	0.13719847
7	-2.69384845	-1.39853119	0.42334584
6	0.68856980	0.09152165	-0.29321512
1	0.93804461	1.19104398	-0.28391837
6	1.26555846	-0.49555435	-1.59289841
1	0.65671714	-1.34141992	-1.90929369
1	1.24878818	0.24303480	-2.39148021
6	2.69171284	-0.94689913	-1.20916711
1	2.79527282	-2.02230184	-1.33588279
1	3.45811910	-0.46498314	-1.81124984
6	2.81811668	-0.56870990	0.26818649
1	3.42449338	-1.26270074	0.84888055
1	3.25249168	0.43859238	0.37208236
7	1.44199422	-0.56661422	0.74702186
6	1.28450373	0.01594467	2.06028383
1	0.24012467	-0.02637995	2.36758362
1	1.87849277	-0.54000651	2.78427248
1	1.61822359	1.06678430	2.07186914
8	1.07369365	2.90039664	0.04533016
1	0.52164923	2.85499658	0.84368642

Conformer 5. ΔE=3.63 kcal mol^{-1}

6	-1.66444769	1.11481396	-0.28354804
1	-1.28337818	2.12522545	-0.39299179
6	-0.77007562	0.05006060	-0.26074602
6	-1.29728257	-1.22608071	-0.09056073
1	-0.64089732	-2.08838915	-0.05649594
6	-2.66543379	-1.37961146	0.03761956
1	-3.11043307	-2.35526327	0.17276776
6	-3.46622659	-0.24500090	-0.01433262
1	-4.54290125	-0.33029963	0.07633928
7	-2.98451180	0.98251799	-0.17085702
6	0.71276201	0.28431125	-0.38679753
1	0.82959115	1.40229149	-0.57646561
6	1.39371421	-0.47324018	-1.53852066
1	0.82536904	-1.37895105	-1.75959794

1	1.43335282	0.12290034	-2.44700810
6	2.76794260	-0.84436429	-0.98001940
1	3.21460223	-1.69969654	-1.48179778
1	3.45057485	0.00221437	-1.05618226
6	2.44591533	-1.10669639	0.48344306
1	1.99236650	-2.10317390	0.61056862
1	3.31407186	-1.05007655	1.14083001
7	1.50489786	-0.04203743	0.79231719
6	0.86441084	-0.08987100	2.08646331
1	0.24814974	0.79815311	2.22589605
1	0.22504241	-0.97272001	2.22196411
1	1.63047415	-0.10120850	2.86189704
8	1.36857677	2.78433437	-0.09336275
1	1.89502616	2.26502980	0.53895190

Conformer 6. ΔE=3.11 kcal mol^{-1}

6	-1.28112804	-1.21311138	-0.04327965
1	-0.60886124	-2.06388089	0.02954728
6	-0.76306788	0.06103285	-0.27422914
6	-1.66358476	1.11346565	-0.34121592
1	-1.29555324	2.12126189	-0.49258630
6	-3.01786360	0.85249025	-0.19740026
1	-3.74885112	1.64679688	-0.24989507
6	-3.42114127	-0.45463304	0.02446764
1	-4.47091162	-0.69432399	0.14527933
7	-2.57219585	-1.47822157	0.10763127
6	0.72174893	0.28349491	-0.41294007
1	0.83361142	1.39009198	-0.65180216
6	1.39923571	-0.52826462	-1.53031450
1	0.82748161	-1.44066636	-1.70923734
1	1.43979237	0.02517893	-2.46545970
6	2.77310006	-0.87813748	-0.95725132
1	3.21419644	-1.75863993	-1.41871543
1	3.46030808	-0.04003390	-1.07504290
6	2.45316596	-1.06974643	0.51744824
1	1.99579978	-2.05670459	0.69210974
1	3.32275996	-0.98469158	1.16976033
7	1.51602545	0.01186079	0.77828816
6	0.87452019	0.01888648	2.07363819
1	0.26084976	0.91380885	2.17600251
1	0.23352682	-0.85617493	2.24458468
1	1.64085150	0.03776399	2.84865567
8	1.25269705	2.82276608	-0.13546417
1	1.81670405	2.32974731	0.48553745

Saddle point R-H10 conformers

Conformer 1. ΔE=9.11 kcal mol^{-1}

6	1.68841519	-1.06734380	-0.63604554
1	1.02355324	-1.78580144	-1.10402295
6	1.19024857	0.19076194	-0.29165683
6	2.06950090	1.07770685	0.30921531
1	1.74361807	2.07015262	0.59168067
6	3.37930133	0.68154409	0.54113732
1	4.08950494	1.34974564	1.00720814
6	3.76269969	-0.59386085	0.16370594
1	4.77665923	-0.93718946	0.33143109
7	2.93528077	-1.46270044	-0.41690737
6	-0.23473118	0.54271243	-0.62257525
1	-0.29324573	0.67083892	-1.70919290
6	-0.81397599	1.82149853	0.02187954
1	-0.46727035	1.92456390	1.04977604
1	-0.51268552	2.71722561	-0.51811896
6	-2.34037156	1.58468140	-0.02114725
1	-2.77766788	1.66090535	0.97296529
1	-2.85982960	2.29422936	-0.66103304
6	-2.45681798	0.15052226	-0.57223334
1	-3.34270032	-0.42366477	-0.07103321
1	-2.66961187	0.13658796	-1.64218262
7	-1.20054955	-0.53078050	-0.30531181
6	-1.11418384	-0.96715489	1.09035088
1	-0.17272095	-1.48650182	1.25430459
1	-1.18148245	-0.14286240	1.81244136
1	-1.93263178	-1.65412337	1.28972248
8	-4.28996324	-1.36218546	0.35195943
1	-4.02741244	-2.06866363	-0.25987188

Conformer 2. ΔE=8.84 kcal mol^{-1}

6	2.07643788	1.05306568	0.29027644
1	1.74851622	2.04957451	0.56286522
6	1.18832270	0.16134681	-0.29854120
6	1.67836062	-1.09749510	-0.63533416
1	1.01324760	-1.81945027	-1.09279563
6	2.99811133	-1.40402620	-0.36548569
1	3.40933088	-2.37357219	-0.60871779
6	3.79613585	-0.43359532	0.22947089
1	4.83602200	-0.63991119	0.45496229
7	3.35333469	0.77495396	0.55168292
6	-0.23361739	0.53147259	-0.62010724
1	-0.29397540	0.66743635	-1.70581618

6	-0.80352054	1.80965962	0.03317538
1	-0.45999257	1.90186780	1.06285484
1	-0.49238110	2.70715631	-0.49787149
6	-2.33175711	1.58555440	-0.01977412
1	-2.77489331	1.66400042	0.97154525
1	-2.84093179	2.30093312	-0.66147984
6	-2.45824397	0.15400230	-0.57582978
1	-3.34797743	-0.41488966	-0.07714406
1	-2.66967272	0.14498722	-1.64609470
7	-1.20690167	-0.53779343	-0.30943942
6	-1.12945331	-0.97584592	1.08671027
1	-0.18658509	-1.49012211	1.25813661
1	-1.20579558	-0.15301858	1.80906406
1	-1.94669042	-1.66645514	1.27862579
8	-4.30465509	-1.34948645	0.34195231
1	-4.06066803	-2.04760182	-0.28682209

Conformer 3. ΔE=0.34 kcal mol^{-1}

6	2.07188344	-1.19088748	-0.02595599
1	1.90001965	-2.26211519	0.02269091
6	0.99166114	-0.32967117	-0.16484825
6	1.26240842	1.03368001	-0.21099755
1	0.44999549	1.74354227	-0.31331610
6	2.57339907	1.46224839	-0.12573429
1	2.82276225	2.51332503	-0.15993036
6	3.57704893	0.50871814	0.00600542
1	4.61525182	0.81183995	0.07594143
7	3.34095336	-0.79665228	0.05723697
6	-0.41555242	-0.85345705	-0.26943256
1	-0.38567019	-1.94786115	-0.14348921
6	-1.12306271	-0.52484138	-1.58445672
1	-0.94030354	0.51965732	-1.84181556
1	-0.78061199	-1.15131530	-2.40481923
6	-2.59116788	-0.72601687	-1.21794548
1	-3.27958881	-0.21708433	-1.88822971
1	-2.83516383	-1.79039089	-1.22104210
6	-2.65171615	-0.18548012	0.20760475
1	-2.97014280	0.91634376	0.16703656
1	-3.38031754	-0.68140776	0.85300595
7	-1.30415871	-0.25644226	0.73027943
6	-1.14717283	-0.76152592	2.07703945
1	-0.10554753	-0.66362755	2.38289676
1	-1.75895044	-0.17993777	2.76531723
1	-1.43539924	-1.81920048	2.16090035
8	-2.47045742	2.36974540	0.15634087
1	-1.69661052	2.02533151	0.63383880

Conformer 4. ΔE=0.88 kcal mol^{-1}

6	1.28390666	0.99423012	-0.28179067
1	0.45588516	1.67978869	-0.43002486
6	1.02589822	-0.36433081	-0.10382634
6	2.11563773	-1.19744521	0.09118560
1	1.96437852	-2.25981458	0.24246238
6	3.39307137	-0.65709680	0.09343459
1	4.26427854	-1.27864818	0.24379553
6	3.53130581	0.70809353	-0.09653744
1	4.51287960	1.16698741	-0.09949370
7	2.49791285	1.52911047	-0.28065003
6	-0.38363659	-0.89255284	-0.14262874
1	-0.35851323	-1.95743415	0.14428664
6	-1.07805735	-0.76074623	-1.49844451
1	-0.90283743	0.24113411	-1.89323817
1	-0.71778198	-1.48821255	-2.22234196
6	-2.54849954	-0.93510045	-1.12380227
1	-3.23203576	-0.50354086	-1.85086588
1	-2.78513555	-1.99579855	-1.02254636
6	-2.63901314	-0.25009303	0.23908463
1	-3.05890969	0.80701410	0.10100799
1	-3.31350393	-0.74367211	0.94468132
7	-1.28387520	-0.15025786	0.73805836
6	-1.10055797	-0.37624735	2.15470711
1	-0.06590655	-0.16962403	2.42768939
1	-1.74200249	0.29771860	2.72116722
1	-1.33845815	-1.41019660	2.44464676
8	-2.73838616	2.28000609	-0.15022404
1	-1.87384257	2.11001615	0.26041608

Conformer 5. ΔE=0.06 kcal mol^{-1}

6	1.62454893	-1.33259820	0.26921979
1	1.41199203	-2.31145716	0.69015900
6	0.60443855	-0.62958389	-0.36963927
6	0.93846002	0.60945027	-0.90526602
1	0.19801905	1.20883414	-1.41889563
6	2.23472731	1.08254692	-0.76754725
1	2.52141283	2.04369374	-1.17052980
6	3.16339128	0.29466005	-0.10329041
1	4.18526530	0.63342504	0.02006537
7	2.87249529	-0.89922212	0.40700370
6	-0.79924924	-1.20128273	-0.40200370
1	-0.71947011	-2.28728100	-0.32014598
6	-1.62019237	-0.78249130	-1.62857532
1	-0.99234379	-0.61457903	-2.50107625

1	-2.31919611	-1.57977083	-1.87036956
6	-2.39342548	0.48159312	-1.18423044
1	-2.04150894	1.38257013	-1.68415244
1	-3.45358528	0.38435864	-1.40765797
6	-2.15600473	0.56718192	0.32622501
1	-1.41456986	1.39171398	0.55854987
1	-3.04000563	0.81998993	0.91272542
7	-1.64513839	-0.72178252	0.69699715
6	-1.11722698	-0.81559687	2.03629725
1	-0.77040193	-1.83067553	2.22972852
1	-0.28179153	-0.12163963	2.21161751
1	-1.90544028	-0.57756708	2.74988067
8	-0.40603115	2.63336636	1.06649454
1	0.40157148	2.09521061	1.11300115

Conformer 6. ΔE=0.00 kcal mol^{-1}

6	0.93031441	0.48000391	-0.99399469
1	0.19683498	1.01817634	-1.58466807
6	0.58018253	-0.70530824	-0.35147767
6	1.58522981	-1.35043609	0.35921176
1	1.37002722	-2.28311834	0.86788417
6	2.85211208	-0.79560633	0.41665093
1	3.65198016	-1.27831318	0.95989807
6	3.07452035	0.40955711	-0.23325570
1	4.04637950	0.88679124	-0.19454963
7	2.13554619	1.04159635	-0.93225244
6	-0.84287128	-1.22655859	-0.32957153
1	-0.80491549	-2.30689317	-0.17459197
6	-1.67550404	-0.85542366	-1.56381456
1	-1.06532698	-0.77787707	-2.46117326
1	-2.41524838	-1.63475429	-1.73158331
6	-2.38013241	0.47052348	-1.18992729
1	-2.01041325	1.31648853	-1.76690579
1	-3.45079937	0.40260598	-1.37081483
6	-2.08935598	0.65089600	0.30195973
1	-1.29066163	1.44902999	0.47045706
1	-2.93505810	0.99981412	0.89571205
7	-1.63952440	-0.63362925	0.75111635
6	-1.08873725	-0.66148276	2.08690756
1	-0.78519556	-1.67754256	2.34048539
1	-0.22848899	0.01197194	2.19831683
1	-1.85593850	-0.34583493	2.79344746
8	-0.26108114	2.56640322	1.06021439
1	0.51877172	2.33547105	0.52819197

Saddle point R-H11 conformers

Conformer 1. ΔE=6.93 kcal mol^{-1}

6	-1.53587311	1.17706899	-0.00459022
1	-0.88284442	2.03417288	0.12661661
6	-0.97123482	-0.07111042	-0.27426394
6	-1.84173839	-1.13806476	-0.43002299
1	-1.46472532	-2.12834357	-0.64915110
6	-3.20758396	-0.92247281	-0.30877036
1	-3.91257665	-1.73311890	-0.42667819
6	-3.65490381	0.35785393	-0.03205085
1	-4.71401159	0.56195555	0.07098292
7	-2.83689939	1.39936260	0.12017974
6	0.52047307	-0.19513890	-0.41770463
1	0.81786793	0.35319809	-1.31824684
6	1.10455277	-1.61042395	-0.53470485
1	0.64193005	-2.27405536	0.19717242
1	0.94330693	-2.04107771	-1.52100600
6	2.59388728	-1.39426523	-0.21025374
1	2.96961202	-2.14439238	0.48420004
1	3.21299596	-1.43883116	-1.10426699
6	2.63703943	0.01827184	0.41651125
1	3.26833148	0.12134853	1.29633499
1	3.06734022	0.73859506	-0.37213096
7	1.27798720	0.43940408	0.68285289
6	0.83423598	0.04761921	2.01893733
1	-0.18062949	0.39789337	2.19301887
1	0.85821109	-1.03768564	2.18467596
1	1.48576185	0.51903659	2.75251031
8	2.95755896	1.91152265	-1.27157947
1	2.26821688	2.29172926	-0.70114663

Conformer 2. ΔE=6.74 kcal mol^{-1}

6	-1.84665962	-1.11080877	-0.41893791
1	-1.46616169	-2.10176356	-0.63753387
6	-0.97005580	-0.04414699	-0.26489194
6	-1.52799468	1.20395075	-0.00036200
1	-0.87821282	2.06093157	0.12919597
6	-2.89972440	1.32510472	0.10627998
1	-3.36410729	2.27862852	0.31443093
6	-3.68015023	0.18693296	-0.06338599
1	-4.75973700	0.24477495	0.01297300
7	-3.17237833	-1.01059879	-0.32393493
6	0.51862889	-0.19052496	-0.41379291
1	0.81738283	0.34557493	-1.32139072

6	1.09082677	-1.61171966	-0.51845189
1	0.62935487	-2.26427953	0.22345595
1	0.91912181	-2.05228157	-1.49838769
6	2.58431446	-1.40134271	-0.20829196
1	2.96073038	-2.14587955	0.49180390
1	3.19630833	-1.46057469	-1.10634777
6	2.64228345	0.01747300	0.40229392
1	3.28197131	0.12646697	1.27532873
1	3.06952436	0.72854985	-0.39753392
7	1.28759073	0.45016091	0.67606486
6	0.85439982	0.06429499	2.01794658
1	-0.16201397	0.40824391	2.19536854
1	0.88577482	-1.01945079	2.19020654
1	1.50764468	0.54427289	2.74450943
8	2.96102738	1.91408660	-1.27674973
1	2.27251053	2.28187052	-0.69742485

Conformer 3. ΔE=0.00 kcal mol^{-1}

6	-1.77266082	1.09041309	-0.73878464
1	-1.27271732	1.90107955	-1.26006698
6	-1.01823688	0.06003594	-0.19145470
6	-1.70160605	-0.94685313	0.48101426
1	-1.14437469	-1.75655544	0.93601483
6	-3.08031280	-0.88188495	0.56686854
1	-3.64625313	-1.64353424	1.08462186
6	-3.73347191	0.19071617	-0.02833995
1	-4.81337223	0.27019379	0.01875327
7	-3.09979947	1.16531217	-0.67109620
6	0.47581906	0.02437672	-0.34710002
1	0.83075769	1.01830738	-0.66385719
6	0.97692955	-1.02928054	-1.34446217
1	0.33898142	-1.91020660	-1.27800386
1	0.93569049	-0.66167335	-2.36703336
6	2.40778743	-1.35398727	-0.86989301
1	2.56014288	-2.42775497	-0.78303790
1	3.16138571	-0.96190644	-1.54909764
6	2.51333188	-0.66025810	0.49019697
1	3.04670279	-1.21682231	1.26131043
1	3.08043922	0.30646588	0.33178776
7	1.16372210	-0.36308634	0.88339053
6	1.06207939	0.59993358	1.95457580
1	0.01846174	0.75704003	2.22310622
1	1.59662119	0.23584249	2.83116730
1	1.49554582	1.56960262	1.65732793
8	3.38873142	1.75560115	-0.50704173
1	3.47672548	2.30026265	0.29155438

Conformer 4. ΔE=0.38 kcal mol^{-1}

6	-1.70226949	-0.88438313	0.52863521
1	-1.13949052	-1.66547123	1.02864539
6	-1.01721515	0.08205510	-0.20679068
6	-1.77091732	1.07588921	-0.81141412
1	-1.27968735	1.85451152	-1.38286385
6	-3.15029980	1.06017552	-0.67287695
1	-3.76828639	1.81876469	-1.13195073
6	-3.72666273	0.04834273	0.07856751
1	-4.80141169	0.00546751	0.21080682
7	-3.02182412	-0.91044773	0.67693578
6	0.47682828	0.01997554	-0.36099391
1	0.84452584	1.00137078	-0.70316966
6	0.95989688	-1.06517763	-1.33364621
1	0.31571659	-1.93837861	-1.23538182
1	0.91238749	-0.72678841	-2.36613183
6	2.39225187	-1.38702189	-0.86181236
1	2.53810304	-2.45918567	-0.74882563
1	3.14335576	-1.01759023	-1.55639357
6	2.51143765	-0.65996840	0.48024683
1	3.04606318	-1.20127467	1.26110982
1	3.07988390	0.29989595	0.29511473
7	1.16626224	-0.34543331	0.87437296
6	1.07658982	0.63962562	1.92660471
1	0.03572368	0.80507394	2.20072023
1	1.61536865	0.28951400	2.80632105
1	1.51254949	1.60033049	1.60548316
8	3.37844524	1.78701543	-0.50691040
1	3.60872948	2.25172770	0.31351767

Conformer 5. ΔE=4.17 kcal mol^{-1}

6	1.98591514	0.86133259	0.85288186
1	1.74341098	1.58248726	1.62840595
6	1.02515646	-0.06728167	0.46186121
6	1.39419420	-0.97268186	-0.52778497
1	0.70084246	-1.73118395	-0.86825712
6	2.66671617	-0.90546087	-1.06920547
1	2.98408157	-1.59628188	-1.83744863
6	3.53834808	0.06790274	-0.60099644
1	4.54167631	0.14709844	-1.00262663
7	3.21278620	0.94005384	0.34769885
6	-0.36187604	-0.01878630	1.06826078
1	-0.29594191	0.53961238	2.00439213
6	-1.00815986	-1.39430733	1.27790021
1	-0.26591137	-2.16570897	1.47189036

1	-1.67111012	-1.34511078	2.13880632
6	-1.82663925	-1.65657736	-0.00758086
1	-1.38724940	-2.43772672	-0.62552426
1	-2.84020471	-1.97141671	0.23158063
6	-1.84172590	-0.31561430	-0.74127854
1	-1.29123438	-0.31814164	-1.69384535
1	-2.91408408	-0.01418216	-1.02914250
7	-1.34430540	0.64667520	0.20332011
6	-0.97729662	1.92833632	-0.36045129
1	-0.65961422	2.60361518	0.43402390
1	-0.16507846	1.85196168	-1.09560972
1	-1.85047699	2.35674284	-0.85268204
8	-4.18794058	0.66018660	-0.63480180
1	-3.77274093	0.94846705	0.19567972

Conformer 6. ΔE=4.41 kcal mol^{-1}

6	1.41056829	-0.96271553	-0.49672534
1	0.71882788	-1.73302503	-0.82027516
6	1.03012066	-0.04539125	0.48203977
6	1.97790746	0.89800909	0.85744082
1	1.73506389	1.63073272	1.61860675
6	3.22705528	0.89260843	0.25986603
1	3.98458485	1.61222883	0.53625453
6	3.48974998	-0.06524338	-0.70794348
1	4.45450619	-0.10142925	-1.19999180
7	2.60271196	-0.98214694	-1.08426067
6	-0.36356725	-0.01951215	1.07863817
1	-0.30830025	0.52916999	2.02146237
6	-1.00029341	-1.40244894	1.27141347
1	-0.25316073	-2.17107690	1.45683412
1	-1.66336547	-1.36684561	2.13294000
6	-1.81694066	-1.65637114	-0.01687010
1	-1.37602058	-2.43268309	-0.63927645
1	-2.83114133	-1.97153458	0.21936216
6	-1.83014974	-0.31100448	-0.74150603
1	-1.26924529	-0.30520708	-1.68762996
1	-2.90113349	-0.01015028	-1.03771170
7	-1.34676998	0.64679872	0.21594287
6	-0.98872206	1.93676548	-0.33360949
1	-0.68639011	2.60901066	0.46982674
1	-0.16944706	1.87611530	-1.06234037
1	-1.86236089	2.35965354	-0.82974815
8	-4.17574868	0.66229704	-0.65085536
1	-3.77239345	0.93592519	0.19029156

Saddle point R-H14 conformers

Conformer 1. ΔE=8.27 kcal mol^{-1}

6	-1.49160988	0.94849020	-0.72756015
1	-0.79764734	1.59692772	-1.25212163
6	-1.05234007	-0.30107222	-0.28587817
6	-1.97038212	-1.09468105	0.38425499
1	-1.69003737	-2.07586398	0.74420330
6	-3.25848616	-0.61922626	0.58525079
1	-3.99775391	-1.21344197	1.10332901
6	-3.58231994	0.63953353	0.10840644
1	-4.57799917	1.04308139	0.24912485
7	-2.71719230	1.41901698	-0.53945081
6	0.35285133	-0.74668503	-0.58492910
1	0.41339902	-0.94085915	-1.66169472
6	0.86635528	-2.00072782	0.14364183
1	0.55360971	-1.97391130	1.18857592
1	0.47980375	-2.91964461	-0.29355923
6	2.40036623	-1.87582207	0.02945790
1	2.87451849	-1.98501582	1.00297574
1	2.82524356	-2.63620061	-0.62230271
6	2.62478927	-0.45666242	-0.55420485
1	3.46097217	0.07881374	-0.11002522
1	2.79194925	-0.51574677	-1.63048619
7	1.37885308	0.28178041	-0.32264592
6	1.34338680	0.83327221	1.00837762
1	0.34904120	1.18094353	1.27576268
1	1.75738259	0.18703613	1.79290058
1	2.03459615	1.76845231	1.01269311
8	2.87610114	2.74634872	0.38523044
1	2.48682297	2.57054355	-0.48744570

Conformer 2. ΔE=7.88 kcal mol^{-1}

6	-1.98058940	-1.08613742	0.32756835
1	-1.70647683	-2.08382035	0.65070846
6	-1.04278376	-0.27211492	-0.29638373
6	-1.46292729	0.99409391	-0.69461262
1	-0.76048531	1.66035582	-1.17977113
6	-2.76576203	1.38298471	-0.44914528
1	-3.12234219	2.36094234	-0.73991458
6	-3.61773369	0.48535090	0.18377676
1	-4.64626638	0.75682951	0.39116850
7	-3.24171112	-0.72869348	0.56559287
6	0.35824167	-0.73697684	-0.58283486
1	0.42344472	-0.93608102	-1.65852908

6	0.85629385	-1.99307417	0.15332478
1	0.53899946	-1.95902853	1.19646581
1	0.46279085	-2.90998709	-0.28127614
6	2.39206073	-1.88395565	0.04260216
1	2.86250800	-1.99416662	1.01778890
1	2.81107678	-2.65114774	-0.60494548
6	2.63294535	-0.46985193	-0.54606706
1	3.47357056	0.05845682	-0.10128106
1	2.80241831	-0.53407565	-1.62165568
7	1.39350862	0.28209548	-0.31943269
6	1.36226950	0.83499379	1.01172985
1	0.36533345	1.16552073	1.29035991
1	1.79488531	0.19624651	1.79161208
1	2.03613710	1.78316741	1.00585174
8	2.84063072	2.77069531	0.34309698
1	2.48468836	2.51553702	-0.52422939

Conformer 3. ΔE=0.00 kcal mol^{-1}

6	-1.67757264	0.66794874	-0.97554884
1	-1.22840879	1.28859344	-1.74568803
6	-0.87001867	-0.16523437	-0.21178265
6	-1.48780448	-0.93213045	0.77001601
1	-0.88684077	-1.57746317	1.39901087
6	-2.85758241	-0.83817465	0.93239201
1	-3.37385892	-1.41558629	1.68634517
6	-3.56830385	0.02275592	0.10384901
1	-4.64304722	0.11887321	0.20545061
7	-2.99760273	0.76830164	-0.83547712
6	0.61112525	-0.23842804	-0.44907338
1	0.90641382	0.60462523	-1.09519363
6	1.09505745	-1.55302811	-1.07606875
1	0.48771490	-2.37500536	-0.69879404
1	0.99456898	-1.53587593	-2.15897497
6	2.55766790	-1.68823568	-0.59881881
1	2.71520485	-2.64895002	-0.11373580
1	3.26673812	-1.61408116	-1.41990806
6	2.74370183	-0.53337301	0.39389814
1	3.33650983	-0.79843430	1.26846134
1	3.22334481	0.32565470	-0.09878251
7	1.38608101	-0.17558526	0.78772761
6	1.28677798	1.06037267	1.49195666
1	0.25593267	1.27137862	1.77410926
1	1.91912531	1.05458986	2.37920428
1	1.65119363	1.91959852	0.83908261
8	1.84254170	2.90262833	-0.46442769
1	0.91743710	3.19720327	-0.45583548

Conformer 4. ΔE=0.56 kcal mol^{-1}

6	-1.46787678	-0.89540779	0.78092134
1	-0.85294867	-1.51098219	1.42903819
6	-0.85849369	-0.15204175	-0.22931034
6	-1.67599954	0.64163125	-1.01876191
1	-1.24291092	1.24381770	-1.80919457
6	-3.04286251	0.65208378	-0.78452038
1	-3.70974932	1.25684489	-1.38258340
6	-3.54137855	-0.13103388	0.24441214
1	-4.60356641	-0.14701477	0.45834346
7	-2.77340547	-0.89239452	1.02259098
6	0.62462015	-0.22910351	-0.45799738
1	0.92141312	0.61119302	-1.10733169
6	1.10862319	-1.54783083	-1.07693219
1	0.50272363	-2.36749100	-0.69283755
1	1.00725491	-1.53822378	-2.15995749
6	2.57202903	-1.67755563	-0.60046709
1	2.73130079	-2.63491925	-0.10956864
1	3.28040978	-1.60708510	-1.42252617
6	2.75667706	-0.51660966	0.38557168
1	3.35073932	-0.77527749	1.26109767
1	3.23515310	0.34027394	-0.11281563
7	1.39917681	-0.15943743	0.77806948
6	1.29535614	1.07688250	1.48003588
1	0.26558274	1.27721794	1.77375338
1	1.93643690	1.07908355	2.36093383
1	1.64226727	1.93680127	0.81995438
8	1.71549609	2.95084387	-0.48708997
1	0.80735882	3.26574716	-0.35191977

Conformer 5. ΔE=5.51 kcal mol^{-1}

6	-1.71314996	-0.99591857	0.90635187
1	-1.30747934	-1.62353305	1.69503488
6	-0.98349121	0.10701705	0.46862092
6	-1.56403588	0.88736983	-0.52591440
1	-1.06186131	1.77298702	-0.89523788
6	-2.80245272	0.53098409	-1.03343700
1	-3.27917072	1.11729771	-1.80616966
6	-3.43044388	-0.59622450	-0.52365482
1	-4.40065270	-0.90205734	-0.89697769
7	-2.90373952	-1.35105569	0.43592421
6	0.39673166	0.37110262	1.03593885
1	0.45041917	-0.09272333	2.02262754
6	0.78174902	1.85591038	1.08299250
1	-0.09035949	2.49474852	1.20526490

1	1.43654057	2.02446667	1.93471725
6	1.54739746	2.12183400	-0.23687781
1	1.01905275	2.81654831	-0.88782319
1	2.52270205	2.55592538	-0.02945055
6	1.69216548	0.74298011	-0.89296988
1	0.94456194	0.60172740	-1.68780697
1	2.67247818	0.56292561	-1.33305630
7	1.47193918	-0.18856802	0.20356429
6	1.37932745	-1.55983724	-0.17546800
1	1.05218569	-2.18023417	0.65685510
1	0.76454943	-1.75018052	-1.06560959
1	2.44311636	-1.92111112	-0.46481038
8	3.89556748	-1.79562622	-0.47337836
1	3.86919544	-1.12571813	0.22955659

Conformer 6. ΔE=5.88 kcal mol^{-1}

6	2.00836427	-0.10937603	-1.27191242
1	1.75455098	-0.06922946	-2.32690613
6	1.03768815	0.18354255	-0.32280582
6	1.41060552	0.09863192	1.01525186
1	0.68684335	0.30878959	1.79478702
6	2.70635214	-0.25975407	1.33462933
1	3.02936461	-0.33916291	2.36297811
6	3.59749101	-0.51841011	0.29885372
1	4.62218675	-0.79781752	0.51413029
7	3.26382654	-0.44685498	-0.98385282
6	-0.36133324	0.57497488	-0.73184042
1	-0.44398167	0.42287434	-1.81271015
6	-0.69224026	2.04209909	-0.37469618
1	-0.04148587	2.36597557	0.43883536
1	-0.52616091	2.71114864	-1.21594504
6	-2.14216045	2.00930840	0.11308084
1	-2.38681085	2.84787557	0.76123209
1	-2.83351472	2.00967306	-0.72981175
6	-2.20393983	0.66466624	0.82187004
1	-1.76599069	0.73275633	1.83122888
1	-3.21241294	0.26228001	0.92690377
7	-1.42250016	-0.18520390	-0.05646700
6	-1.24320826	-1.54131841	0.32446209
1	-0.50694526	-2.03636255	-0.30504424
1	-0.98669266	-1.68408284	1.38339424
1	-2.23553879	-2.10129656	0.19012899
8	-3.66011705	-2.19987576	-0.35043001
1	-3.46747401	-1.47618977	-0.96888086

Conformer 7. ΔE=5.63 kcal mol^{-1}

6	-1.57983911	0.87205066	-0.49117671
1	-1.08177255	1.76874001	-0.84486731
6	-0.98343793	0.08027847	0.48955434
6	-1.69665563	-1.03548181	0.91089697
1	-1.28640634	-1.67761892	1.68197148
6	-2.93100325	-1.31440478	0.34980220
1	-3.50880459	-2.17233892	0.66305460
6	-3.41819263	-0.45992381	-0.62826945
1	-4.37921906	-0.64570363	-1.09317382
7	-2.76167650	0.61864827	-1.04496366
6	0.40023310	0.36448508	1.04244407
1	0.46625141	-0.09448524	2.03091165
6	0.77410012	1.85266479	1.08203190
1	-0.10091150	2.48611188	1.20977903
1	1.43437019	2.02639813	1.92856326
6	1.52834941	2.12094252	-0.24369567
1	0.99031718	2.81065086	-0.89150637
1	2.50313549	2.55986611	-0.04393531
6	1.67460115	0.74186306	-0.89844452
1	0.91763362	0.59461927	-1.68274700
1	2.65078963	0.56737882	-1.34980931
7	1.47377551	-0.18858151	0.20393001
6	1.39217251	-1.56185802	-0.16858352
1	1.07986819	-2.18194900	0.66988191
1	0.77178364	-1.76311208	-1.05241290
1	2.45742514	-1.91445307	-0.46397411
8	3.90816358	-1.78079421	-0.46954559
1	3.87357145	-1.09191479	0.21450680

Conformer 8. $\Delta E=5.00$ kcal mol^{-1}

6	-1.79305438	1.21625167	-0.13835255
1	-1.26332176	2.16196866	-0.16826172
6	-1.09075465	0.03982484	-0.40872400
6	-1.80218987	-1.14668485	-0.33208187
1	-1.32147099	-2.09667759	-0.52148190
6	-3.15068920	-1.11194511	-0.00211574
1	-3.72964710	-2.02186280	0.06914107
6	-3.74439547	0.11477140	0.23567371
1	-4.79556826	0.17917440	0.49109221
7	-3.08179847	1.26954170	0.16977655
6	0.38208235	0.14012490	-0.76962285
1	0.46647437	0.64977661	-1.73101415
6	1.13998565	-1.19287355	-0.75293231
1	0.60513517	-2.01349671	-1.22491184
1	2.07968110	-1.06793052	-1.28822467
6	1.41640132	-1.41294255	0.74264596

1	0.67122370	-2.06989397	1.19016466
1	2.39870383	-1.84975096	0.89982391
6	1.31608736	0.00801389	1.36045871
1	0.48340435	0.06220956	2.06214255
1	2.22329694	0.29935192	1.88615646
7	1.07020782	0.94282329	0.25196825
6	2.25650085	1.59515117	-0.22121519
1	2.03413219	2.21318873	-1.09079865
1	2.67656822	2.22192584	0.56450144
1	3.05982195	0.86291889	-0.52388870
8	4.25223143	-0.34705999	-0.24972383
1	4.89500570	0.26087208	0.14873303

REFERENCES

(1) Zheng, J.; Yu, T.; Papajak, E.; Alecu, I. M.; Mielke, S. L.; Truhlar, D. G. Practical Methods for Including Torsional Anharmonicity in Thermochemical Calculations on Complex Molecules: The Internal-Coordinate Multi-Structural Approximation. *Phys. Chem. Chem. Phys.* **2011**, *13* (23), 10885–10907. https://doi.org/10.1039/C0CP02644A.

(2) Zheng, J.; Truhlar, D. G. Quantum Thermochemistry: Multistructural Method with Torsional Anharmonicity Based on a Coupled Torsional Potential. *J. Chem. Theory Comput.* **2013**, *9* (3), 1356–1367. https://doi.org/10.1021/ct3010722.

(3) Zheng, J.; Bao, J. L.; Zhang, S.; Corchado, J. C.; Meana-Paneda, R.; Chuang, Y.-Y.; Coitino, E. L.; Ellingson, B. A.; Truhlar, D. G. Gaussrate17. University of Minnesota: Minneapolis, MN, 2017.

(4) Zheng, J.; Bao, J. L.; Meana-Paneda, R.; Zhang, S.; Lynch, B. J.; Corchado, J. C.; Chuang, Y.-Y.; Fast, P. L.; Hu, W.-P.; Liu, Y.-P.; Lynch, G. C.; Nguyen, K. A.; Jackels, C. F.; Fernandez-Ramos, A.; Ellingson, B. A.; Melissas, V. S.; Villa, J.; Rossi, I.; Coitino, E. L.;

Pu, J.; Albu, T. V. Polyrate-Version 2016-2A. University of Minnesota: Minneapolis, MN, 2016.

(5) Yu, H. S.; He, X.; Truhlar, D. G. MN15-L: A New Local Exchange-Correlation Functional for Kohn–Sham Density Functional Theory with Broad Accuracy for Atoms, Molecules, and Solids. *J. Chem. Theory Comput.* **2016**, *12* (3), 1280–1293. https://doi.org/10.1021/acs.jctc.5b01082.

(6) Dunning, T. H. Gaussian Basis Sets for Use in Correlated Molecular Calculations. I. The Atoms Boron through Neon and Hydrogen. *J. Chem. Phys.* **1989**, *90* (2), 1007–1023. https://doi.org/10.1063/1.456153.

(7) Zhao, Y.; Truhlar, D. G. The M06 Suite of Density Functionals for Main Group Thermochemistry, Thermochemical Kinetics, Noncovalent Interactions, Excited States, and Transition Elements: Two New Functionals and Systematic Testing of Four M06-Class Functionals and 12 Other Function. *Theor. Chem. Acc.* **2008**, *120* (1), 215–241. https://doi.org/10.1007/s00214-007-0310-x.

(8) Bao, J. L.; Zheng, J.; Truhlar, D. G. Kinetics of Hydrogen Radical Reactions with Toluene Including Chemical Activation Theory Employing System-Specific Quantum RRK Theory Calibrated by Variational Transition State Theory. *J. Am. Chem. Soc.* **2016**, *138* (8), 2690–2704. https://doi.org/10.1021/jacs.5b11938.

(9) Bao, J. L.; Zhang, X.; Truhlar, D. G. Predicting Pressure-Dependent Unimolecular Rate Constants Using Variational Transition State Theory with Multidimensional Tunneling Combined with System-Specific Quantum RRK Theory: A Definitive Test for Fluoroform Dissociation. *Phys. Chem. Chem. Phys.* **2016**, *18* (25), 16659–16670.

https://doi.org/10.1039/C6CP02765B.

(10) Kee, R. J.; Coltrin, M. E.; Glarborg, P.; Zhu, H. *Chemically Reacting Flow*, 2nd ed.; Kee, R. J., Coltrin, M. E., Glarborg, P., Zhu, H., Eds.; Wiley Online Books; John Wiley & Sons, Inc., 2017. https://doi.org/doi:10.1002/9781119186304.

(11) Russo, P.; Cesario, A.; Rutella, S.; Veronesi, G.; Spaggiari, L.; Galetta, D.; Margaritora, S.; Greenberg, P. G. and D. S. Impact of Genetic Variability in Nicotinic Acetylcholine Receptors on Nicotine Addiction and Smoking Cessation Treatment. *Current Medicinal Chemistry*. 2011, pp 91–112. https://doi.org/http://dx.doi.org/10.2174/092986711793979715.

(12) Poling, B. E.; Prausnitz, J. M.; O'Connell, J. P. *Properties of Gases and Liquids, Fifth Edition*; McGraw-Hill Education: New York, 2001.

(13) Grajales-González, E.; Monge-Palacios, M.; Sarathy, S. M. Collision Efficiency Parameter Influence on Pressure-Dependent Rate Constant Calculations Using the SS-QRRK Theory. *J. Phys. Chem. A* **2020**, *124* (31), 6277–6286. https://doi.org/10.1021/acs.jpca.0c02943.

(14) Grajales-González, E.; Monge-Palacios, M.; Sarathy, S. M. A Theoretical Study of the Ḣ- and HOȮ-Assisted Propen-2-Ol Tautomerizations: Reactive Systems to Evaluate Collision Efficiency Definitions on Chemically Activated Reactions Using SS-QRRK Theory. *Combust. Flame* **2021**, *225*, 485–498. https://doi.org/https://doi.org/10.1016/j.combustflame.2020.11.015.

(15) Troe, J. Predictive Possibilities of Unimolecular Rate Theory. *J. Phys. Chem.* **1979**, *83* (1), 114–126. https://doi.org/10.1021/j100464a019.

(16) Gilbert, R. G.; Luther, K.; Troe, J. Theory of Thermal Unimolecular Reactions in the Fall-off Range. II. Weak Collision Rate Constants. *Berichte der Bunsengesellschaft für Phys. Chemie* **1983**, *87* (2), 169–177. https://doi.org/10.1002/bbpc.19830870218.

(17) Carstensen, H. H.; Dean, A. M.; Green, N. J. B.; Carr, R. W. *The Kinetics of Pressure-Dependent Reactions*; Green, N. J. B., Carr, R. W., Eds.; Elsevier: Amsterdam, The Netherlands, 2007; Vol. 42.

(18) Chang, A. Y.; Bozzelli, J. W.; Dean, A. M. Kinetic Analysis of Complex Chemical Activation and Unimolecular Dissociation Reactions Using QRRK Theory and the Modified Strong Collision Approximation. *Z. Phys. Chem.* **2000**, *214*, 1533–1568. https://doi.org/10.1524/zpch.2000.214.11.1533.

(19) Senosiain, J. P.; Klippenstein, S. J.; Miller, J. A. Reaction of Ethylene with Hydroxyl Radicals: A Theoretical Study. *J. Phys. Chem. A* **2006**, *110* (21), 6960–6970. https://doi.org/10.1021/jp0566820.

(20) Borduas, N.; Murphy, J. G.; Wang, C.; Da Silva, G.; Abbatt, J. P. D. Gas Phase Oxidation of Nicotine by OH Radicals: Kinetics, Mechanisms, and Formation of HNCO. *Environ. Sci. Technol. Lett.* **2016**, *3* (9), 327–331. https://doi.org/10.1021/acs.estlett.6b00231.

(21) Borduas, N. The Atmospheric Fate of Organic Nitrogen Compounds. Ph.D. Dissertation, University of Toronto, Toronto, 2015.
